# Water quality management could halve future water scarcity cost-effectively in the Pearl River Basin

**DOI:** 10.1038/s41467-024-49929-z

**Published:** 2024-07-06

**Authors:** Safa Baccour, Gerwin Goelema, Taher Kahil, Jose Albiac, Michelle T. H. van Vliet, Xueqin Zhu, Maryna Strokal

**Affiliations:** 1 Department of Agricultural Economics, Finance and Accounting, University of Cordoba, 14071 Cordoba, Spain; 2 Independent researcher, Groningen, The Netherlands; 3 Water Security Research Group, Biodiversity and Natural Resources Program, International Institute for Applied Systems Analysis (IIASA), 2361 Laxenburg, Austria; 4 Department of Economic Analysis, University of Zaragoza, 50009 Zaragoza, Spain; 5 Department of Physical Geography, Faculty of Geosciences, Utrecht University, 3584CS Utrecht, The Netherlands; 6 Environmental Economics and Natural Resources, Wageningen University, 6708PB Wageningen, The Netherlands; 7 Earth Systems and Global Change, Wageningen University, 6708PB Wageningen, The Netherlands

**Keywords:** Pollution remediation, Water resources, Computational science, Element cycles

## Abstract

Reducing water scarcity requires both mitigation of the increasing water pollution and adaptation to the changing availability and demand of water resources under global change. However, state-of-the-art water scarcity modeling efforts often ignore water quality and associated biogeochemical processes in the design of water scarcity reduction measures. Here, we identify cost-effective options for reducing future water scarcity by accounting for water quantity and quality in the highly water stressed and polluted Pearl River Basin in China under various socio-economic and climatic change scenarios based on the Shared Socio-economic Pathways (SSPs) and Representative Concentration Pathways (RCPs). Our modeling approach integrates a nutrient model (MARINA-Nutrients) with a cost-optimization procedure, considering biogeochemistry and human activities on land in a spatially explicit way. Results indicate that future water scarcity is expected to increase by a factor of four in most parts of the Pearl River Basin by 2050 under the RCP8.5-SSP5 scenario. Results also show that water quality management options could half future water scarcity in a cost-effective way. Our analysis could serve as an example of water scarcity assessment for other highly water stressed and polluted river basins around the world and inform the design of cost-effective measures to reduce water scarcity.

## Abstract (web summary)

Future water scarcity is expected to increase up to fourfold in most parts of the Pearl Basin by 2050, driven by changes in both water quantity and quality. Water quality management options could cost-effectively half future water scarcity.

## Introduction

Global water scarcity challenges the achievement of the Sustainable Development Goals (SDGs)^1,2^. Water scarcity is a result of both quantity and quality changes^3^. Over half of the global population lives in areas where water is limited (not enough) or/and too polluted^4^. This challenges the balance between water demand and supply^5^, and causes economic risks to water use sectors such as energy, agriculture, households, and industries^6^. In addition, water pollution, for example, high nitrogen (N) concentrations, can lead to eutrophication and health issues. Water pollution in rivers results often from intensive food production^7^ and mismanaged urban^8,9^ systems. The challenge is how to mitigate water scarcity in a cost-effective way^3,10,11^ to ensure sufficient water of good quality to fulfill human, environmental, social, and economic demands and support sustainable development^12,13^.

Assessments of water scarcity management focuses mostly on water quantity and overlook water quality^5,14^. Cost-effective options to reduce future water scarcity associated with both quality and quantity are hardly explored while considering biogeochemistry in the river system. A wide range of water scarcity indexes has been employed such as the Falkenmark (FLK), water stress (WSI), and criticality ratio^15^. The WSI is known as the ratio of water use to water availability^5^. Other studies use the concept of greywater footprint^16^ which determines the amount of water needed to dilute pollutants in wastewater to meet the water quality standard. Following this concept, an innovative water scarcity indicator has been developed by van Vliet et al.^3^, considering water quantity and quality, environmental flow requirements, and water use for different sectors. This indicator has been used to assess global water scarcity with an expansion of clean water technologies^17^. However, an integrated biophysical-economic assessment of how to reduce water scarcity associated with water quality and quantity under various future climate and socio-economic scenarios in cost-effective ways is still lacking.

Previous studies have mentioned the importance of addressing water quantity and quality management challenges using economic instruments, including water pricing and pollution tax^18,19^. Strokal, et al.^18^ present an integrated modeling approach to identify cost-effective options for reducing coastal eutrophication. This modeling approach is built on the MARINA-Nutrients model (version 1.0, Model to Assess River Inputs of pollutaNts to seAs) and a cost-optimization procedure. MARINA-Nutrients simulates river export of nutrients in dissolved inorganic and organic forms from human activities while considering biogeochemistry, climate change, and socio-economic developments at the sub-basin scale. The model considers the export of nutrients from land to rivers and by rivers to seas. During this export, biogeochemical processes associated with retention and losses of nutrients are accounted for. Integrating MARINA-Nutrients with a cost-optimization procedure enabled to identify cost-effective options to reduce coastal eutrophication. However, this integrated approach has not been applied to water scarcity problems and did not consider management options for reducing water scarcity.

This paper presents an integrated modeling approach combining biophysical (i.e., MARINA-Nutrients model (version 2.0) for river export of total dissolved N) and economic (i.e., water scarcity cost optimization) modeling at the river basin scale to identify cost-effective combinations of management options for reducing river export of total dissolved N (TDN) and improving water supply to reduce water scarcity, while accounting for climate and socio-economic changes and sub-basin characteristics (e.g., urbanization, land use). This integrated modeling approach is built on the approach of Strokal et al.^18^, but extended to water scarcity including options for water quality and quantity management and accounting for biogeochemical interactions between them. We calculate water scarcity for the future in which we account for water quality (i.e., TDN-related) and quantity, following the approach of van Vliet, et al.^3^. Future trends are based on a combination of RCP (Representative Concentration Pathway) and SSP (Shared Socio-economic Pathway) scenarios (see “Methods” for details). This integrated modeling approach is applied to the Pearl River Basin, a highly water stressed and polluted basin in China, which is also experiencing rapid climate and socio-economic changes.

## Results

### Future annual and seasonal water scarcity

**Fig. 1:**
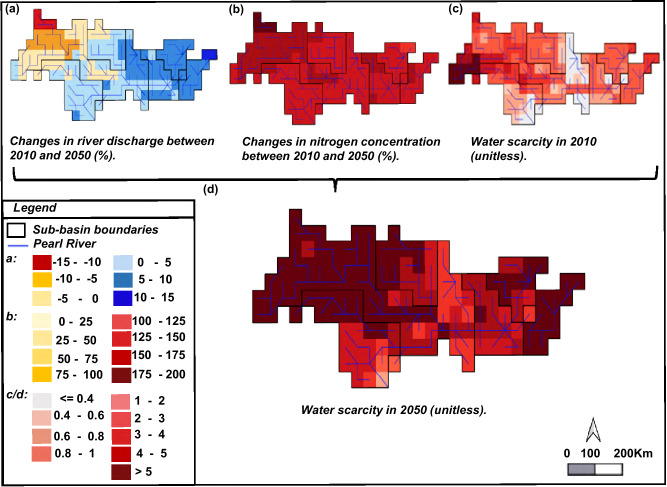
Future water scarcity. **a** Changes in annual river discharge between 2010 and 2050 under RCP8.5- SSP5 in the Pearl River Basin at 0.5° resolution. **b** Changes in annual nitrogen concentrations in rivers between 2010 and 2050 under RCP8.5-SSP5 in the Pearl River Basin at 0.5° resolution. **c** Annual water scarcity in 2010 in the Pearl River Basin at 0.5° resolution. **d** Annual water scarcity in 2050 under RCP8.5- SSP5 in the Pearl River Basin at 0.5° resolution. SSP is short for Shared Socio-economic Pathway. RCP is short for Representative Concentration Pathway. Source: The data for river discharges are from the VIC model^2^.

Water scarcity in the Pearl River Basin is expected to increase by a factor of 4 by 2050 compared to 2010 (Fig. 1c, d). This increase is calculated for the whole basin under the RCP8.5-SSP5 scenario. Water scarcity was about 1.2 in 2010 with substantial variability throughout the year. This number is much >0.4 (threshold) indicating high water scarcity^17^. In 2050, water scarcity is expected to become around 5 under RCP8.5-SSP5. High water scarcity is calculated for most grid cells in this scenario (Fig. 1). Increased future water scarcity is associated with the combined effects of growing water withdrawals, climate change impacts on water availability, and high TDN concentrations. The river discharge of the whole Pearl River Basin is projected to increase by 4% for RCP8.5 between 2010 and 2050. However, projected changes in river discharge vary spatially, for example, it is projected to decrease in the North-West (e.g., −15%) and increase in the South-East (+15%) (Fig. 1a). This spatial variation is caused by increased precipitation in the southern part of the basin and decreased precipitation in the northern part combined with changes of human and land use activities. The total water use is estimated to increase by 44% in 2050 under SSP5. This rise in water use is mainly related to the development of industrial activities with a relative decrease in agricultural and domestic water use.

**Fig. 2:**
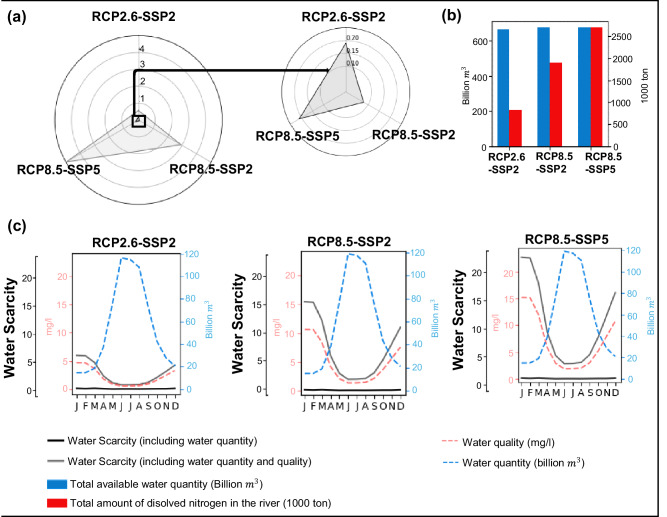
Seasonal future water scarcity. **a** Future annual water scarcity including water quantity, and water quantity and quality, **b** water quantity (total available water in the river) and quality (total amount of dissolved nitrogen in the river), and (**c**) seasonal future water quantity, water quality, water scarcity including water quantity, and water scarcity including and quality in 2050 by months, and climate and socio-economic scenarios (RCP2.6-SSP2; RCP8.5-SSP2; RCP8.5-SSP5). The total available water is the river discharge. The total amount of nitrogen in the river includes nitrogen from all sources (diffuse and point). RCP is short for Representative Concentration Pathway. SSP is short for Shared Socio-economic Pathway. Source: The total available water (discharge) is from the VIC model^2^. River pollution with nitrogen is calculated with nitrogen data from the MARINA-Nutrients model (version 2.0)^35^ combined with discharge data from the VIC model ^2^. Water scarcity is calculated using river discharges and nitrogen pollution in rivers according to ref. ^3^ (Supplementary note 2). In **a** and **c**, the black color represents water scarcity including only water quantity and the gray color shows water scarcity including both water quantity and quantity. In **b**, **c**, the blue schemes represent river discharge whereas the red represents the water quality parameter *N*. In **a**, the small black triangle in the radar chart indicates that water scarcity including only water quantity reaches by 0.22 in all scenarios and it is zoomed in the second radar chart. This value is multiplied by a factor of 24 when considering water quality in the estimation of water scarcity.

Reductions in the supply of clean water are projected in 2050, driven by the increase of TDN pollution levels in rivers of the Pearl Basin (Supplementary Figs. 2 and 3). Average yearly TDN concentrations are estimated to increase by 135% in RCP8.5-SSP5 during 2010–2050. This higher concentration results in higher values in the winter months induced by high impact of climate change and strong growth of economic activities under the RCP8.5-SSP5 scenario. The spatial distribution of TDN concentrations across grid cells of the Pearl River Basin is high (Fig. 1b). Almost all areas do not meet the *N* quality standard (1.0 mg/l) for all sectors in 2050 for the RCP8.5-SSP5 scenario. Water pollution is considered the strongest driver of exacerbating water scarcity levels in the Pearl River Basin. Annual water scarcity considering only water quantity is expected to reach around 0.2, whereas considering both water quality and quantity is expected to be a factor of 24 higher compared to water scarcity considering only water quantity in 2050 under the RCP8.5-SSP5 scenario (Fig. 2a). This demonstrates the importance of integrating water quality in assessing water scarcity. Figure 2b shows a small change in the total available water quantity in all scenarios. However, there is a considerable increase in TDN in rivers in the RCP8.5-SSP2 and RCP8.5-SSP5 scenarios compared to the RCP2.6-SSP2 scenario. While there is no considerable shift in river discharge at the basin scale across the different scenarios, the fact that water quality changes substantially implies that water scarcity issues are more strongly associated with water quality rather than water quantity. The disparity between RCP8.5-SSP2 and RCP8.5-SSP5 scenarios shows the impact of socio-economic activities on water scarcity levels in 2050. Future projections show a 70% increase in annual water scarcity level (2.9-5) including both water quality and quantity in 2050 under the RCP8.5-SSP5 scenario compared to RCP8.5-SSP2. The gap between RCP2.6-SSP2 and RCP8.5-SSP2 scenarios demonstrates how climate change will affect water scarcity levels in 2050. Future projections indicate a slight difference in future water scarcity including only water quantity between the RCP2.6-SSP2 and RCP8.5-SSP2 scenarios because water availability is almost the same. However, the annual water scarcity level including water quantity and quality rises by a factor of five (0.6-2.9) in 2050 under the RCP8.5-SSP2 scenario compared to the RCP2.6-SSP2. The reason is the high nitrogen concentration under the severe climate scenario RCP8.5, brought on by the impending land use change and rising future emissions.

The large variability of water scarcity levels throughout the year is mainly driven by TDN pollution (Fig. 2c). Water scarcity including both water quantity and quality is higher in all months, with variability throughout the year compared to the water scarcity level including only water quantity. Water scarcity based on water quantity only is estimated to be 0.1 per month in 2050 under all climate and socio-economic scenarios, illustrating that water quantity was not a considerable driver of water scarcity in the Pearl River Basin. However, water pollution with TDN has a considerable impact on increasing water scarcity in the Pearl River Basin during the winter and spring seasons. The seasonal analysis of water scarcity suggests that low water scarcity is projected to occur during the summer months (May-Sept) and high water scarcity to occur during the winter and spring months of the year (Oct-Apr) in 2050 under all climate and socio-economic scenarios. The low water scarcity during the summer is explained by the increase in water quantity due to the high discharges (June-Aug), and low TDN concentrations in rivers of the Pearl Basin during the summer months. However, low river discharges in the winter months (Dec-Mar) and a high level of TDN concentration due to lack of water for dilution leads to high levels of water scarcity.

Results indicate that water scarcity is considerably larger in 2050 under the severe climate and socio-economic scenario (RCP8.5-SSP5) compared to the RCP8.5-SSP2 and RCP2.6-SSP2 scenarios. The reason is the impact of climate change and the development of socio-economic activities, especially in the industrial sector, that lead to higher levels of water use and TDN pollution. The increase in water availability is largely offset by the absolute increase in water use under future climate and socio-economic scenarios. Under the RCP2.6-SSP2 scenario, monthly TDN concentrations meet water quality standards for domestic water use (May-Sept) and industrial and agricultural water use (April-Oct). However, under RCP8.5-SSP2 and RCP8.5-SSP5 scenarios, the TDN concentrations meet the water quality standard (1 mg/l) for industrial and agricultural water use just from June to August of the year, but not for domestic water use in the whole year.

### Cost-effective options to reduce future water scarcity

All water quantity and quality management options assessed could contribute to reducing future water scarcity (Tables 1 and 2). Results indicate that the water quantity management option in agriculture “low-pressure pipe irrigation” reduces water use by around 11 billion m^3^ at a cost of about $0.25 billion under all scenarios. Water quantity management options in households “Water saving from bathroom faucet, kitchen faucet, showerhead, and washing machine” reduce water use by only 1–2 billion m^3^ at $17-78 billion depending on the scenario. Infrastructure improvement options such as water storage and transport options are crucial because they increase water supply under future climate conditions. Water storage could considerably increase water availability by 164-212 billion m^3^ depending on the scenario, but it requires high investment and operating costs under all climate and socio-economic scenarios that range from $118 to 153 billion. Water transport also increases water supply by 14-56 billion m^3^ depending on the scenario but at a lower cost compared to water storage (Table 1). Water quality management options reduce TDN pollution by 574 kton in the severe climate and socio-economic scenario RCP8.5-SSP5 at a cost of only $0.3 billion (Table 2).

**Table 1:** Total costs and abatements of water quantity management options under different climate and socio-economic scenarios in 2050

Management options		RCP2.6-SSP2		RCP8.5-SSP2		RCP8.5-SSP5	
Sector	Water quantity options	Costs ($ billion)	Water saving (billion m^3^)	Costs ($ billion)	Water saving (billion m^3^)	Costs ($ billion)	Water saving (billion m^3^)
**Agriculture**	Low-pressure pipe irrigation	0.25	11	0.25	11	0.25	11
**Households**	Water-saving bathroom faucet	2	0.09	1	0.04	2	0.09
	Water-saving kitchen faucet	2	0.43	2	0.43	2	0.42
	Water saving showerhead	11	0.74	11	0.74	11	0.73
	Water-saving washing machine	38	0.54	3	0.05	63	0.9
**Water infrastructure improvement**	Water storage	153	212	118	164	118	164
	Water transport	3	56	1	17	1	14

The information on water savings and costs are used for estimating total available water in the river and water scarcity with policy implementation in 2050, water scarcity abatement compared to the baseline scenario (2010), and cost-efficiency, contributing to the development of the MACC curve.

RCP is short for Representative Concentration Pathway. SSP is short for Shared Socio-economic Pathway. Source: Results of the cost-optimization analysis (Supplementary Note 2; Supplementary Table 3).

**Table 2:** Total costs and abatements of water quality management options under different climate and socio-economic scenarios in 2050

Management options		RCP2.6-SSP2		RCP8.5-SSP2		RCP8.5-SSP5	
Sector	Water quality options	Costs ($ billion)	N reduced (kton)	Costs ($ billion)	N reduced (kton)	Costs ($ billion)	N reduced (kton)
**Sources of nitrogen input to the river**	Apply synthetic nitrogen fertilizer on land	0.25	118	0.25	206	0.25	213
	Recycle manure as slurry on land	0.0002	9	0.0002	92	0.0002	144
	Treat human waste with tertiary technologies	0.00003	128	0.00003	140	0.00003	217

The information on N reduced and costs is used for estimating the total amount of dissolved nitrogen in the river and water scarcity with policy implementation in 2050, water scarcity abatement compared to the baseline scenario (2010), and cost-efficiency, contributing to the development of the MACC curve.

RCP is short for Representative Concentration Pathway. SSP is short for Shared Socio–economic Pathway. Source: Results of the cost-optimization analysis (Supplementary Note 2; Supplementary Table 3).

**Fig. 3:**
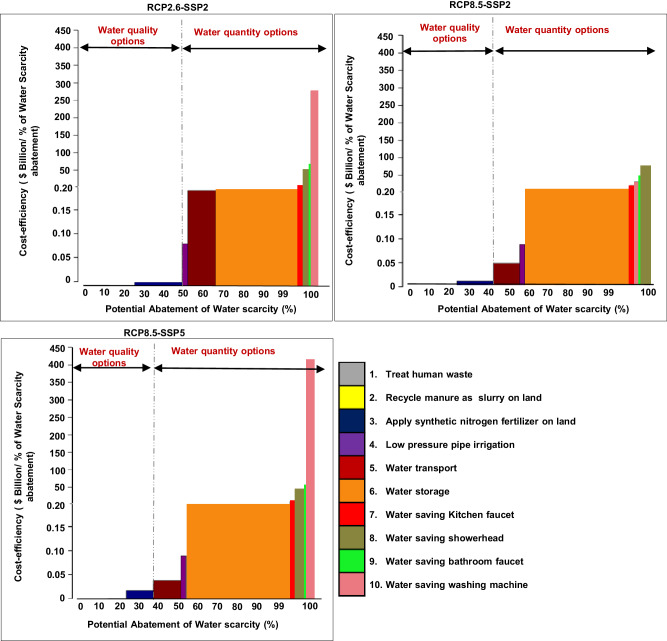
Marginal Abatement Cost Curve (MACC) for water quantity and quality management options in 2050 by climate and socio-economic scenarios (RCP2.6-SSP2; RCP8.5-SSP2; RCP8.5-SSP5). RCP is short for Representative Concentration Pathway. SSP is short for Shared Socio-economic Pathway. Source: Water scarcity is calculated using river discharges and nitrogen pollution in rivers according to ref. ^3^. The cost-efficiency is calculated based on the cost-optimization model that indicates the total annual costs of cost-effective water scarcity mitigating management options, and the water scarcity abatement of management options. Example: The water transport option reduces water scarcity by 14% with a cost-efficiency of 0.2 $Billion/1% of water scarcity under the RCP2.6-SSP2 scenario. In other words, water transport costs 0.2 $ billion to reduce 1% of water scarcity.

To compare the cost-efficiency of the different water quantity and quality management options in reducing water scarcity, we constructed the Marginal Abatement Cost Curve (MACC) of the future water scarcity management options, highlighting their water scarcity abatement potential and cost-efficiency (Fig. 3). Results indicate that water quality management options provide around 50% of the water scarcity abatement potential in the RCP2.6-SSP2 scenario and around 40% in the RCP8.5-SSP2 and RCP8.5-SSP5 scenarios at a low cost (i.e., $0.01 billion/1% of water scarcity abatement). Household water quantity management options “water saving from bathroom faucet, kitchen faucet, showerhead, and washing machine” are the least cost-effective water scarcity management options, with an abatement potential of <1% and high costs of $14-450 billion/1% of water scarcity abatement in all climate and socio-economic scenarios. The water storage option has the highest abatement potential among the water quantity options in terms of reducing water scarcity, with an abatement of 33-44% depending on the scenario and costs of $2-4 billion/1% of water scarcity abatement in all climate and socio-economic scenarios. These results highlight that water quality management options are the most cost-effective in reducing water scarcity in the Pearl River Basin and should be prioritized when designing a water scarcity management strategy. Investing in wastewater treatment plants is the most cost-effective management option. Recycling manure as slurry on land reduces water scarcity by 10% in the RCP8.5-SSP5 at a low cost.

Combining the potential abatement options for water quality and quantity in all sectors (agriculture and households) represents a cost-effective way to address water scarcity. The reductions in water scarcity by agricultural water quantity options attain 5% at a cost of about $0.25 billion in 2050 for all climate and socioeconomic scenarios (a cost-efficiency of $0.05 billion/1% of water scarcity abatement). However, reductions in water scarcity when addressing both the quantity and quality of water in agriculture could achieve 30% under the RCP2.6-SSP2 and RCP8.5-SSP2 scenarios and 26% under the RCP8.5-SSP5 scenario at a cost of $0.49 billion in 2050 (a cost-efficiency of $0.02 billion/1% of water scarcity abatement). A slight reduction of water scarcity by 1% is found for household water quantity options at a high cost of about $53 billion for the RCP2.6-SSP2, $17 billion for the RCP8.5-SSP2, and $79 billion for the RCP8.5-SSP5 scenario. Combined household water quantity and quality options reduce water scarcity by 25% at a cost of about $53 billion for the RCP2.6-SSP2 (a cost-efficiency of $2 billion/1% of water scarcity abatement), by 14% at a cost of $17 billion for the RCP8.5-SSP2 (a cost-efficiency of $1.3 billion/1% of water scarcity abatement), and by 15% at a cost of $79 billion for the RCP8.5-SSP5 scenario (a cost-efficiency of $5 billion/1% of water scarcity abatement). Combining the management of water quantity and quality is more cost-effective for agriculture than for households.

## Discussion

This paper provides an integrated analysis of future water scarcity abatement in the Pearl River Basin under climate and socio-economic change scenarios, highlighting the cost-efficiency of water quantity and quality management options. Our results show that water scarcity will increase under the future scenarios with spatial and seasonal variabilities. The intensification of water scarcity due to water pollution is in line with previous water scarcity studies in China^17,19,20^. Van Vliet et al.^17^ indicated that the percentage of people affected by water scarcity globally is higher when water quality is taken into account (40%) rather than solely water quantity (30%). Strokal et al.^18^ estimated that 3$ billion are needed to reduce coastal eutrophication at the river mouth of the Yangtze basin in 2050 using a cost-optimization procedure. This can be done by implementing water quality management options for nitrogen and phosphorus (e.g., recycling manure, treating human waste). The cost-optimization analysis of our paper indicates that only 0.3 $ billion is needed to reduce by half water scarcity in the Pearl basin by implementing water quality options in 2050 for the RCP8.5-SSP5 scenario. Water quality management options are the most cost-effective in highly water stressed and polluted river basins such as the Pearl under future climate and socio-economic change scenarios. Recycling manure as slurry on land is often suggested as an important measure to reduce water pollution and improve water quality^21–23^. Manure recycling to cropland is an effective measure for drastically reducing coastal eutrophication^18^ and improving soil quality^24–26^. Reducing the use of synthetic fertilizer is also highlighted as a cost-effective measure to reduce nitrate concentration in the Ebro basin, improving both water and atmosphere qualities at negative costs^27,28^. These win-win management options may encourage farmers’ endorsement and could be the focus of future more detailed follow-up research.

Our study contributes to existing knowledge in several aspects. First, an integrated MARINA-cost optimization modeling approach that considers both water quantity and quality management options is developed to identify cost-effective options for reducing water scarcity under various future climate and socio-economic change scenarios. Such options include mitigation of water pollution sources on land (e.g., less use of synthetic fertilizers, and more manure recycling) and adaptation to changing availability of water resources (e.g., water-saving technologies). The effects of these options are combined to estimate river export of TDN (considering the biogeochemical cycling of N from land to sea and associated retention and losses) and associated water scarcity in the basin. This integrated approach was applied by Strokal et al.^18^ to reduce coastal eutrophication, but did not consider water quantity management options and water scarcity. However, this integrated approach to water scarcity that considers various water quantity and quality management options has not been applied in prior research. The MACC analysis has been mostly used in climate policymaking, guiding greenhouse gas mitigation measures. MACC has also been used in water quantity related challenges^29,30^, but only a few studies have applied it to water scarcity that integrates both quality and quantity in a spatially explicit way for the future^31^. The MACC provides a ranking of water scarcity management options and insights on least-cost combinations of agriculture and households water quantity and quality management options. Lastly, our modeling approach could be useful for assessments at different spatial and temporal scales and for other pollutants, which could also be applied to other river basins in the world. Although our results demonstrate a reasonable level of agreement with other modeling studies, it is important to indicate that our modeling approach also involves some uncertainties. Those are related to the MARINA-Nutrients model structure, inputs, and aggregations. Model structure uncertainties arise from simplified land-to-sea nutrient flows in which intra-annual legacy effects are disregarded. Another simplification is the equalized amount of TDN throughout the year that could affect future TDN concentrations and future seasonal water scarcity. We downscaled sub-basin values of TDN to grids and months as explained in Supplementary note 1, introducing uncertainties. This was required to estimate pollution at the grid of the sub-basin outlet. However, our main conclusions are based on sub-basin analyses and cost-effective measures. Despite the uncertainties, the MARINA-Nutrients model has been evaluated using a “building trust” approach^32^, which includes validation, sensitivity analysis, and comparison with other studies. Such evaluation was for different nutrient forms and across China including the Pearl River Basin. A sensitivity analysis of dissolved inorganic nitrogen (DIN) and dissolved organic nitrogen (DON) river exports for 2012 has been conducted by Wang et al.^33^ and revealed that river exports are relatively susceptible to changes in river discharge, manure excretion, and direct discharges of animal manure. The model was evaluated across Chinese rivers^33–36^ including seasons^37^ and lakes^38–40^ and in other regions in the world^18,41,42^, demonstrating that the MARINA-Nutrients model is reliable with acceptable performance in quantifying river export of nutrients. Most of the model inputs are taken from peer-reviewed studies and published models (Supplementary Table 4). River discharge was derived from simulations by the VIC hydrological model, considered the most reliable source for China^1,43–45^. Water use is derived from the PCR-GLOBWB global hydrological model, which has been evaluated and validated in different studies^46–48^.

Uncertainties in our estimates may originate from several aspects. One of them is that we did not explicitly consider the legacy effects^49^. These may influence nutrient cycling and water quality^50,51^. The legacy effects across inter-annual variations entail that past actions or events such as historical land use practices have long-term consequences on nutrient pollution levels. Nutrient legacies from previous fertilizer applications or land management practices can persist in the soil and affect nutrient availability to plants over multiple growing seasons. Thus, the legacy effects further challenge the goal of improving water quality^52^. Inefficient management practices and land use change could intensify the impacts of climate-induced droughts or floods and lead to increased water scarcity^53^. Addressing nutrient legacies requires a combination of measures that mitigate the adverse legacy effects and work towards achieving sufficient clean water resources for future generations.

Another source of uncertainties is that we did not consider other nutrients such as phosphorus, which may influence our water quality results and their effects on water scarcity. Phosphorus can stay even longer in the system because of its strong binding ability (compared to nitrogen) and can be released into water over time for decades. In our earlier study^54^, we assessed the legacy effect of phosphorus on the river export of phosphorus on a large scale. One of our findings was that phosphorus was released from the soil continuously into rivers even after stopping the use of fertilizers on land. However, river export of phosphorus was only slightly affected. The main reason was that the dominant source of phosphorus in many rivers was the sewage system compared to agricultural runoff. In our study area, the dominant sources differ among the sub-basins^36,55^. In the downstream sub-basins, the sewage systems are important because of the urbanization trends. In those sub-basins, the biases in our water quality simulation associated with legacy effects is somewhat lower. In the up-middle stream sub-basins, agriculture plays an important role. Here we may under- or over-estimate pollution levels depending on soil characteristics and hydrology. For river exports of nitrogen, this may be different and likely also depends on the dominant sources. Furthermore, nitrogen is strongly connected to air and microbial activities. Some nitrogen can easily leave the system via denitrification processes and thus it is perceived as more mobile than phosphorus. As a result, nitrogen to phosphorus ratios may also change because of the legacy effects and affect water quality^56^.

We believe that the uncertainties do not change largely our main conclusions. This is because the aim of this paper is solely on nitrogen focusing on the long-term perspectives (2010–2050) rather than intra-annual variabilities and highlight the need for proactive and forward-thinking water management policies and regulations. Policymakers could use our insights for their long-term policy plans when developing measures to mitigate the effects of climate and socio-economic changes on future water scarcity. A more detailed modeling approach (e.g., at monthly level) would be required to account for the intra-annual variability in climate, hydrology and socio-economic effects on water scarcity estimates including other nutrients.

Additionally, uncertainties arise also from the data used in the assumptions of SSPs-RCPs scenarios and the costs of water management options. Considering reactive environmental protection with considerable globalization and open market in the RCP8.5-SSP5 scenario could be challenging to achieve in 2050. However, the different scenarios assessed provide a wide range of conceivable changes in society and climate, along with future water scarcity. Cost data for management options used in the cost-optimization procedure are derived from studies carried out in other regions and basins worldwide (Supplementary Table 4) because of the limited data in the Pearl basin. Despite all uncertainties, our model provides a better understanding of future annual and seasonal water scarcity and cost-effective water management options, assisting policymakers in determining the feasibility and robustness of water quantity and quality management alternatives for addressing water scarcity.

We also performed a sensitivity analysis to better understand how uncertainties in costs and water scarcity levels would influence our main conclusions. This is because the costs of water management options can be highly variable depending on factors that include technological, political and environmental conditions. In our paper, the water quality management options were identified to be the most cost-effective to reduce water scarcity. To test the sensitivity of our cost-efficiency calculations to cost assumptions, we increased the costs of the water quality management options by +50% and +100%, while we kept the costs of the water quantity management options as in the original model run. We then recalculated the cost-efficiency of all the management options and compared them under three climate and socio-economic scenarios (SSP2-RCP2.6, SSP2-RCP8.5 and SSP5-RCP8.5) and different levels of water scarcity (low (0.1), medium (0.2) and high (0.4)). This sensitivity analysis resulted in 12 extra model runs and the results are presented in Supplementary Table 6. They show that changes in the costs of the water quality management options did not affect largely our main conclusions that water quality management options are the most cost-effective to reduce future water scarcity. This conclusion remained the same for all three scenarios even when we doubled the costs of the water quality management options (+100%). The same applies for changed water scarcity levels. These results indicate the robustness of our main conclusions. However, these results are for a set of options that we considered and assessed in this paper, which are considered the most relevant to address water quantity and quality management issues worldwide including in our case study.

Simplified assumptions have been applied to our modeling approach especially for the scenarios. Including only TDN as a water quality constituent to effectively demonstrate how its presence and pollution can directly affect water scarcity concerns. Future works would include other water quality constituents such as other nutrients (P), chemicals (e.g., painkillers, antibacterial agents), and salinity^3,18^ in the assessment of water scarcity, which could increase levels of water scarcity due to the additional amount of water that will be needed for dilution to obtain sufficient water quality levels. Considering the indirect effects that could occur between different management options (e.g., evaporation and water losses are not considered for water storage and water transport options), could improve the estimation of future water scarcity and its management. Moreover, including other water quantity and quality management options that span different sectors such as water reuse, rainwater harvesting^11^, desalination plants, and treated wastewater reuse^3^ could improve the cost-efficiency analysis and increase the abatement potential of water scarcity^57^. Despite these limitations, our modeling approach generates useful insights to mitigate water scarcity and identify cost-effective water quantity and quality options to meet the SDGs, specifically, SDG targets 6.3 and 6.4, which aim to improve water quality and increase water use efficiency^22^.

## Methods

### Study case

The Pearl River Basin is China’s second largest river basin, with a drainage area of 450,000 km^2^. It has three major tributaries: Xijiang, Dongjiang, and Beijiang. Xijiang is the biggest sub-basin with a length of about 2075 km and a drainage area of 353000 km^2^ which accounts for three-quarters of the total drainage area of the Pearl River Basin. It has a subtropical and tropical monsoon climate with a dry season from October to March and a rainy season from April to September. The Pearl River Basin, especially the Delta region, is economically developed and is of great importance in the socio-economic development of China. High climate variability and the high level of socio-economic development make this basin a potential region prone to water scarcity, and a valuable case study area for investigating the management options to mitigate water scarcity.

### An integrated modeling approach for water scarcity

**Fig. 4:**
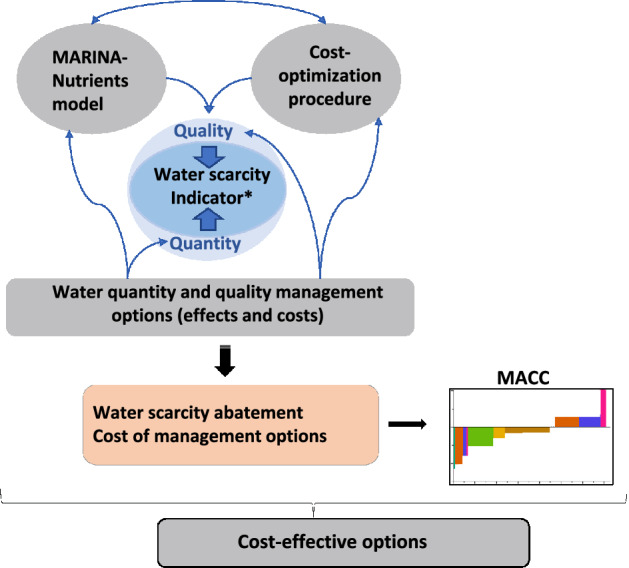
A conceptual framework of our integrated modeling approach for mitigating water scarcity while accounting for quality and quantity. MARINA-Nutrients is short for a Model to Assess River Inputs of pollutAnts to seAs. MACC is the Marginal Abatement Cost Curve. *Water scarcity indicator is calculated using river discharges and N pollution in rivers according to ref. ^3^.

Our modeling approach integrates the MARINA-Nutrients model (version 2.0) with a cost- optimization procedure and the Marginal Abatement Cost Curve (MACC) method to identify cost-effective options to reduce future water scarcity (Fig. 4). Several versions of MARINA exist and have been applied, evaluated, and validated for China^33^ and worldwide^18^. In this study, we use the Chinese version of the model (version 2.0), which is an updated version of Strokal, et al.^35^ for river export of TDN (total dissolved nitrogen consisting dissolved inorganic and organic forms)^33^. A cost-optimization procedure is then used to calculate the potential and cost of several water quantity and quality management options to save water and reduce N pollution, respectively. Water scarcity is calculated using the approach of van Vliet, et al.^3^. The MACC method is used to rank the management options based on their cost-efficiency to reduce water scarcity. Below, we first explain the water scarcity indicator. Next, we describe the MARINA-Nutrients model and its integration with the cost-optimization procedure, as well as the MACC method and the climate and socio-economic scenarios.

### Water scarcity indicator

The water scarcity indicator of Van Vliet et al.^3^ is used to calculate water scarcity on different spatial and temporal scales, considering concurrently water quantity, water quality, environmental flow requirements, and water use of different sectors. This indicator is defined as the ratio of water withdrawals of acceptable quality to the total water availability. Per sector it includes the required water withdrawals, and the amount of water that is needed for dilution to reach water of acceptable and useable quality for a certain sector^3^. The indicator is unitless and indicates what part of the available water is used. Therefore, it is interpreted as if water scarcity exceeds 1, there is not enough water available to supply human water demands with water of sufficient quality. The calculation of water scarcity indicator is presented in Eq.1.The numerator represent the acceptable quality water withdrawals and includes the required water withdrawals $({D}_{j,m}-{{TS}}_{j,m})$, where ${D}_{j,m}$ is water demand and ${{TS}}_{j,m}$ is water saving for each sub-basin j and month m, as well as the amount of water required for dilution$\,{{dq}}_{j,m}$ to achieve acceptable and usable water quality per sector and spatial location (i.e., concentrations levels below the maximum threshold concentration for intended sectoral water use). The denumerator represents the total water availability and is the total available water discharge in sub-basin ${{Qa}}_{j,m}$ minus the total amount of water for the environmental flow requirements ${{EFR}}_{j,m}$. ${N}_{j.m}$ is the nitrogen concentration in sub-basin j in month m and ${{N}_{\max }}_{j.m}$ is the water quality standard for nitrogen concentration.

1 $${{WSq}}_{j,m}=\frac{\left({D}_{j,m}-{{TS}}_{j,m}\right)+\,{{dq}}_{j,m}}{{{Qa}}_{j,m}-\,{{EFR}}_{j,m}}$$

2 $${{dq}}_{j,m}=\left\{\begin{array}{ll} 0 \hfill & {N}_{j.m}\le {N}_{\max }\,\hfill \\ \frac{{{Qa}}_{j.m}\,*\,{N}_{j.m}}{{{N}_{\max }}_{j.m}}-{{Qa}}_{j.m} \hfill & {N}_{j.m}\ge {N}_{\max }\,\end{array}\right.$$

Annual and monthly water scarcity is calculated for the Pearl River Basin for 2010 (baseline) and 2050 on different spatial scales: the whole river basin, and for every grid cell of 0.5°×0.5° spatial resolution (Supplementary note 1). For 2050 water scarcity is calculated for three different combined climate and socio-economic scenarios: RCP2.6-SSP2, RCP8.5-SSP2, and RCP8.5-SSP5, to cover a large range of uncertainties in future socio-economic developments and climate change. Water scarcity is calculated using river discharges (to indicate renewable water availability) and N pollution (i.e., TDN concentration relative to threshold concentration) in rivers according to van Vliet, et al.^3^ (Supplementary note 2). The discharge is from the VIC (Variable Infiltration Capacity) model^1^ (Supplementary Note 3). The total amount of dissolved N in the river is calculated with N data from the MARINA-Nutrients model (version 2.0)^33^. Water use is defined as the net human water consumption which is equal to water withdrawn from rivers minus the return flow. Monthly water use data for the year 2010 is derived from the PCR-GLOBWB global hydrological model for two socio-economic scenarios (SSP2 and SSP5)^58^. Water use in 2050 is calculated by multiplying the calculated sectoral water use in 2010, by projected sectoral water use changes in the Pearl River Basin between 2010 and 2050. Sectoral changes between 2010 and 2050 in both the SSP2 and SSP5 scenarios are derived for the domestic, industrial, and agricultural sectors from Yao et al.^59^. Supplementary Table 4 displays model inputs and their spatial and temporal variability.

The MARINA-Nutrients model (version 2.0) quantifies river export of TDN in dissolved inorganic (DIN) and organic (DON) forms by source and sub-basin for 1970, 2000, and 2050. In this study, we select TDN as a water quality constituent because it often plays a central role in the nutrient dynamics of rivers and sea and is a key driver of eutrophication, which leads to problems like oxygen depletion in coastal waters. TDN impacts are also spread on several sectors including agriculture, domestic, and nature. The quantification of TDN river export is carried out in three steps. First, TDN inputs including DIN (dissolved inorganic N) and DON (dissolved organic N) from diffuse and point sources to surface waters are quantified. For diffuse sources, the calculations are done as a function of runoff and nutrient inputs to land corrected for crop uptake and soil retention. For point sources, the calculations include population, treatment efficiencies in wastewater treatment plants, and connection rates to sewage systems. Second, TDN export to sub-basin outlets is quantified considering retentions in and losses from rivers (denitrification for DIN, damming, and water consumption for all nutrient forms). Third, TDN export from sub-basin outlets to the river mouth is quantified considering losses during export^33^. The model has been validated and evaluated by Wang, et al.^33^ (see Supplementary Note 2).

Nutrient inputs to rivers result from diffuse (anthropogenic and natural) and point sources. Diffuse anthropogenic sources include inputs of nutrients to rivers through runoff from the application of animal manure (for DIN, DON), synthetic fertilizers (for DIN, DON), and human waste (for DIN, DON) on agricultural land, atmospheric N deposition on agricultural land (for DIN), biological N_2_ fixation by crops (for DIN), and leaching of organic matter from agricultural land (for DON). Diffuse natural sources include inputs of nutrients to rivers through runoff from atmospheric N deposition on non-agricultural land (for DIN), biological N_2_ fixation by natural vegetation (for DIN), and leaching of organic matter from non-agricultural land (for DON). The point sources of nutrients in rivers are direct discharges of animal manure (untreated, for DIN and DON) and human waste (untreated, for DIN and DON), and sewage effluents (after treatment, for DIN and DON) from the population connected to sewage systems. In our paper, TDN is the sum of DIN and DON exports by rivers from all sources.

### Water scarcity cost-optimization model

**Fig. 5:**
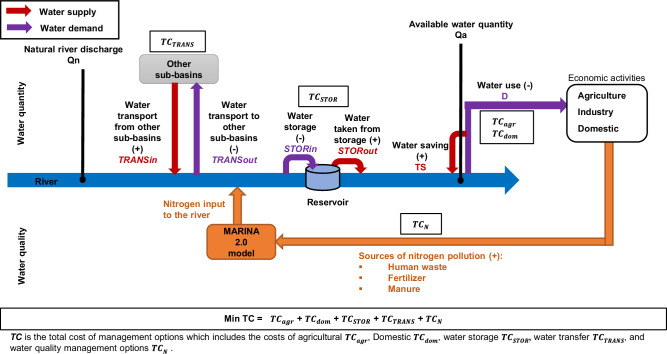
An overview of the water scarcity management options in the water balance of a sub-basin, together with the connections between the cost functions and the mitigation of water scarcity (quality and quantity). The dark blue middle line represents the main river. The red lines represent the flow of water entering the river and the purple lines represent the flow out of the river due to the implementation of water quantity management options. The difference between the natural river discharge on the left (***Qn***) and the available water quantity (***Qa***) on the right reflects the combined impact of the water quantity management options on water availability in the river. The orange lines represent the flow of nitrogen from the economic activities to the river. The white box at the bottom is the objective function of the cost- optimization model. The other white boxes show the different cost functions with different water scarcity management options. (Supplementary note 2).

The integrated modeling approach is developed to determine the most cost-effective combinations of water quantity and quality management options to mitigate water scarcity under different climate and socio-economic scenarios (RCP-SSP), (Fig. 5) using the optimization software: General Algebraic Modeling System (GAMS)^60^. The water scarcity cost-optimization model includes an objective function and a set of constraints. The objective function is to minimize the total cost (TC) of management options for mitigating water scarcity in the Pearl River Basin in 2050 under climate change and socio-economic developments. Costs of the different water management options are connected to mitigate water scarcity by altering the available water quantity and the quality of water, which depend on the water saving and the N reduced of management strategies. The total cost of management options is the sum of the costs of the different types of management options, including water-efficient agricultural practices (*TC*_*agr*_), domestic technologies (*TC*_*dom*_), water storage (*TC*_*STOR*_), water transfer (*TC*_*TRANS*_), and water quality management (*TC*_*N*_). Several constraints are considered in the model including the level of water scarcity, water quantity options, river export of DIN and DON from sub-basins and sources, and nutrient management options (Supplementary note 2).

Water scarcity is constrained for each sub-basin and river month under different levels. The optimization procedure is conducted for water scarcity levels of 0.4 (high water scarcity), 0.2 (medium water scarcity), and 0.1 (low water scarcity). If these levels cannot be reached simultaneously, the lowest possible water scarcity is set as the constraint. The level of water scarcity depends on the total amount of water needed to satisfy sectoral demands and the amount of water needed for dilution to reach sufficient water quality (i.e., concentration below water quality standard or threshold concentration) in each sub-basin and each river month. The input values of DIN and DON and the TDN concentrations are derived from the MARINA-Nutrients model (version 2.0). The water quality standard related to TDN concentration used in this study is derived from the Chinese water quality classes and described in the national environmental quality standards for surface water^61^. Water quality standards for nitrogen concentration are respectively 1.0, 1.5, and 2.0 mg/l for the domestic, industrial and agricultural sectors^61^. In this study, the water quality standard is 1.0 mg/l for the domestic sector, which is considered the most stringent standard and is therefore also sufficient for the industrial and agricultural sectors^62^. This choice implies that if the standard for the domestic sector is met, then water quality is also sufficient for the industrial and agricultural sectors. Selecting the strictest standards has also benefits for the other sectors. Our water scarcity cost-optimization model provides the cost and the potential in saving water or reducing TDN pollution of each water management option, respecting the level of water scarcity and the water quality standards. This information is used to generate the Marginal Abatement Cost Curve (MACC) by estimating the water scarcity abatement and the cost-efficiency of each management strategy.

### Water scarcity management options

Several water quantity and quality management options for reducing water scarcity are considered in this study. The Marginal Abatement Cost Curve (MACC) method^63^ is used to present and order these different management options based on their cost-efficiency showing the potential of each option in reducing water scarcity. The MACC was developed using the simulation results of the water scarcity cost-optimization model and shows the cost of reducing water scarcity by one additional unit (expressed in % of water scarcity). This information reveals the most cost-effective management options (cheapest) in reducing water scarcity (Supplementary Note 4; Supplementary Fig. 1). The abatement level of management options is related to water savings or reduction of N pollution.

Water quantity management options include several water-efficient agricultural and domestic technologies and water transfer options (Supplementary Table 3). Agricultural water quantity management options focus on the efficiency of irrigation water use addressing water scarcity and raising water saving while the domestic water quantity management options focus on implementing water-efficient faucets in kitchens and bathrooms, together with water-efficient showerheads, toilets, washing machines, and dishwashers. The costs and water savings for agricultural and domestic sectors are derived from several studies^64–66^. Water transfer options have a significant potential to increase water supply and include water storage and water transport between sub-basins. The stored water in reservoirs can be moved from the relative abundance of summer months to the relative scarcity of winter months. The cost for water storage is derived from Grygoruk et al.^67^ (Supplementary Note 5; Supplementary Tables 1–2). The selected water management options are feasible to be implemented in China and in the Pearl River Basin. The implementation costs of different alternatives are determined from river basins and regions with similar climate conditions because of the lack of data in the Pearl River.

Water quality management options are identified for diffuse and point sources to reduce river export of the total dissolved N (TDN = DIN + DON) from sub-basins (Supplementary Table 3). Management options for diffuse sources reduce the N of synthetic fertilizers and increase the recycling of manure in cropland as organic fertilizer (slurry or solid). The implementation of manure recycling depends on several factors, including the distance to the manure storage area, the type of fertilizer, and application techniques. Zhang et al.^68^ indicate that farmers perceived barriers to using recycled livestock manure in China, and there is a need for a transparent manure transfer and accurate information on the composition and prices of manure products. Management options for point sources include treating manure and human waste with primary, secondary, and tertiary technologies, which reduce the direct discharge of untreated substances into rivers. The costs for mitigation of diffuse and point management options are taken from Strokal, et al.^18^.

### Climate and socioeconomic scenarios

The integrated modeling framework was run for the baseline in 2010 and for three scenarios combining the RCP (radiative forcing of 2.6 and 8.5 W/m^2^)^69^ and SSP^70^ (SSP2 and SSP5) scenarios in 2050: RCP2.6-SSP2, RCP8.5- SSP2, and RCP8.5-SSP5. The three scenarios are possible combinations of the RCP and SSP^33^. The RCP2.6-SSP2 represents a scenario with low climate change and business-as-usual economic activities. The RCP8.5-SSP2 represents a scenario with high climate change and business-as-usual economic activities. The RCP8.5-SSP5 represents a scenario with high climate change and high economic growth. The SSP2 is considered the business-as-usual scenario with intermediate challenges for adaptation and mitigation of climate change and a medium level of economic and technological developments and population growth. However, the SSP5 represents economic optimism, with rapid economic and technological developments and low population growth.

### Sensitivity analysis

A set up of a sensitivity analysis demonstrates how uncertainty in the cost of water quality management options would influence the cost-efficiency and the abatement potential of the water quantity and quality management options. To do so, we run the water scarcity cost-optimization model for different values of the cost of the water quality management options (base value; +50%; and +100% of the unit cost; see Supplementary Table 5) under the SSP2-RCP2.6, SSP2-RCP8.5, and SSP5-RCP8.5 scenarios and different water scarcity levels of 0.4 (high water scarcity), 0.2 (medium water scarcity), and 0.1 (low water scarcity). The base cost values of the water quantity management options are kept constant to see how the cost-efficiency ratios of all the management options and their ranking change if the water quality management options would be more expensive under the different levels of water scarcity and the various RCP-SSP scenarios.

### Reporting summary

Further information on research design is available in the Nature Portfolio Reporting Summary linked to this article.

### Supplementary information

Supplementary Information

Peer Review File

Reporting Summary

## Data Availability

The data supporting the findings in this study are available within the paper and its Supplementary Information. All data sources are provided in this paper.

## Code availability

All equations are available in the Supporting information in this paper.
