# Supplementary material for: Water quality management could halve future water scarcity cost-effectively in the Pearl River Basin
Source: Nat Commun. 2024 Jul 6;15:5669. doi: 10.1038/s41467-024-49929-z (PMC11227540; doi:10.1038/s41467-024-49929-z)
Supplement: Supplementary file 1 — Supplementary Information [file 41467_2024_49929_MOESM1_ESM.pdf]

Supplementary Information

Water quality management could halve future water scarcity cost-effectively in the Pearl River Basin

Supplementary notes:

- Supplementary note 1. Downscaling of annual nitrogen river exports at the sub-basinscale to the monthly and grid scales.
- Supplementary note 2. Mathematical formulation.
- Supplementary note 3. Water availability (VIC hydrological model)
- Supplementary note 4. The Marginal Abatement Cost Curve (MACC).
- Supplementary note 5. Water transport management option.

Supplementary tables:

- Supplementary Table 1. Cost of transporting water in China's South-North water transfer project for different cities, and distance of the cities from the source region (Dengzhou).
- Supplementary Table 2. Transport distances between the different sub-basins of the Pearl River Basin (thousand km).
- Supplementary Table 3. Effects and costs of water quantity and quality management options.
- Supplementary Table 4. Data sources and their temporal and spatial variability.
- Supplementary Table 5. Set-up of a sensitivity analysis for costs of water quantity and quality management options.
- Supplementary Table 6. Sensitivity analysis results under the SSP-RCP scenarios, water scarcity level, and increased cost of water quality management options.

Supplementary figures:

- Supplementary Figure 1. Marginal abatement cost Curve (MACC).
- Supplementary Figure 2. Monthly nitrogen concentrations in the rivers of the Pearl River sub-basins in 2010 and in 2050 for the RCP2.6-SSP2, RCP8.5-SSP2, and RCP8.5-SSP5 scenarios (mg/l).
- Supplementary Figure 3. Changes in nitrogen concentration in the rivers of the Pearl River basin between 2010 and 2050 for the RCP2.6-SSP2, RCP8.5-SSP2, and RCP8.5-SSP5 scenarios at 0.5° x 0.5° grid (%).

Supplementary references

Supplementary note 1. Downscaling annual nitrogen river exports at the sub-basin scale to the monthly and grid scales.

Total Dissolved Nitrogen (TDN) concentration (C) is used in this study as an indicator for water quality. TDN concentrations are calculated by dividing the total amount of dissolved nitrogen (inorganic and organic) in the river (kton) by the natural river discharge (billion m^3). This is done for the Pearl River Basin, the sub-basins of the Pearl River, and all of the 0.5° x 0.5° grid cells in the Pearl River Basin. The total amount of dissolved nitrogen in the river is derived from the MARINA-Nutrients model (Model to Assess River Inputs of Nutrients to seAs, version 2.0) [1]. The MARINA-Nutrients model quantifies the total annual dissolved nitrogen (inorganic and organic) per sub-basin. The total annual dissolved nitrogen is derived for the six sub-basins of the Pearl River and the whole Pearl River Basin for the years 2012 and 2050. The nitrogen input data for the year 2012 is used for the year 2010. The total annual dissolved nitrogen is downscaled to the total monthly dissolved nitrogen by dividing the total annual dissolved nitrogen by twelve months. Here it is assumed that the amount of dissolved nitrogen concentrations are equal for all months of the year.

The TDN is downscaled from the sub-basin scale to 0.5° x 0.5° grid scale. Thisis done in three steps. First, the TDN that comes to the river from activities within the sub-basin is calculated. This is done because TDN in a sub-basin includes DIN (dissolved inorganic N) and DON (dissolved organic N) from upstream sub-basins. TDN is the sum of DIN and DON. The amount of TDN that comes from activities within the sub-basin is calculated by subtracting TDN in that sub-basin from TDN from adjacent upstream sub-basins. Secondly,the TDN in a sub-basin is divided by the number of 0.5° x 0.5° grid cellsin that sub-basin. The number of 0.5° x 0.5° grid cells in the sub-basins of the Pearl River are derived from the ddm30 river basin map [2]. This average value of TDN in a sub-basinis given to each cell in that sub-basin. In this calculation, it is assumed that the TDN inputs to the river are equally distributed within the sub-basin and are therefore equal for all cells in a sub-basin. Thirdly, the TDN in a cell is calculated. This is calculated as TDN inputs to the river in that cell plus the sum of TDN inputs to the river of all the upstream cellsin that sub-basin (e.g., mimicking the routing scheme for the cells within the sub-basin). The direction of the cells is determined with the ddm30 drainage direction map derived from [2].

Supplementary note 2. Mathematical formulation.

An overview of the main equations of the integrated modeling approach is presented in this subsection. The modeling approach entails integrating a biophysical (MARINA-Nutrients) model for nitrogen [1] with a cost optimization procedure for abating water scarcity, accounting for future climate change and socio-economic development. MARINA-Nutrients quantifies river export of nitrogen by sources as a function of human activities (population, urbanization, fertilizer application) and land characteristics (land use, hydrology), accounting for nutrient losses and retentions in soils. We used an updated version of MARINA-Nutrients model (version 2.0) developed by Wang et al. [1].The approach of Strokal et al. [3] that integrates the MARINA-Nutrients model (version 1.0) with a cost-optimization approach to reduce coastal eutrophication in the Yangtze Basin was followed and adjusted for reducing water scarcity in this study. Supplementary Box 1 presents the equations of the integrated modeling approach.

Supplementary Box 1. Overview of integrating the MARINA-Nutrients model (version 2.0) and a cost-optimization procedure to minimize the total costs of management options in 2050 to mitigate future water scarcity. This method is inspired by the approach of [3]. The cost-optimization procedure is used to identify the cost-effective water quantity and quality management options to reduce nutrient pollution and increase water supply. MARINA-Nutrients is used to quantify river export of nutrients.

MARINA 2.0-Water scarcity cost optimization model

Objective function Minimize the total cost of management options to mitigate water scarcity.

$$min\ TC = TC_{agr} + TC_{dom} + TC_{STOR} + TC_{TRANS} + TC_{N} \quad \text{(Eq. 1)}$$

$$\begin{cases} TC_{agr} = \sum_j^6 \{\sum_m^{12} [\sum_{Oagr}^3 (Da_{j.m} * (1 - Sa_{Oagr}) * Xa_{j.Oagr} * C_{Oagr})]\} \\ TC_{dom} = \sum_j^{12} [\sum_{Odom}^6 (Xd_{j.Odom} * P_j * C_{Odom})] \\ TC_{STOR} = \sum_j^6 [\sum_m^{12} (STORin_{j\,m} * C_{STOR})] \\ TC_{TRANS} = \sum_j^6 \{\sum_{j2}^6 [\sum_m^{12} (TRANSin_{j\,.j2\,.m} * C_{TRANS.\,j.\,j2})]\} \\ TC_N = \sum_j^6 [\sum_{Odif}^3 (\frac{Xdif_{Odif\,.\,j}}{CFdif_{Odif}} * C_{Odif}) + \sum_{Opnt}^8 (\frac{Xpnt_{Opnt\,.\,j}}{CFpnt_{Opnt}} * C_{Opnt}] \end{cases}$$

Subject to

$$WSq_{j.m} \leq WSq_{max} \quad \text{(Eq. 2)}$$

Water quantity management options

$$Qa_{j.m} = Qn_{j.m} - STORin_{j.m} + STORout_{j.m} + \sum_{j2=1}^{6}(TRANSin_{j.j2.m}) - \sum_{j2=1}^{6}(TRANSout_{j.j2.m}) + \sum_{i=j+1}^{n} TS_{j2.m} \quad \text{(Eq. 3)}$$

$$\sum_{m=1}^{12} STORin_{j.m} = \sum_{m=1}^{12} STORout_{j.m} \quad \text{(Eq. 4)}$$

$$\sum_{j=1}^{6}[\sum_{j2=1}^{6}(TRANSout_{j.j2.m})] \leq \sum_{j=1}^{6} Qa_{j.m} \quad \text{(Eq. 5)}$$

$$TS_{j.m} = \sum_{Oagr=1}^{3}(Da_{j.m} * Xa_{j.Oagr} * Sa_{Oagr}) + \sum_{Odom=1}^{6}(Sd_{Odom} * Xd_{j.Odom} * P_j \quad \text{(Eq. 6)}$$

$$\sum_{Oagr=1}^{3} Xa_{j.Oagr} \leq 1 \quad \text{(Eq. 7)}$$

Water scarcity

$$WSq_{j.m} = \frac{(D_{j.m} - TS_{j.m}) + dq_{j.m}}{Qa_{j.m} - EFR_{j.m}} \quad \text{(Eq. 17)}$$

$$EFR_{j.m} = Qa_{j.m} * 0.37 \quad \text{(Eq. 18)}$$

$$dq_{j.m} = \begin{cases} 0 & ;\ N_{j.m} \leq N_{max} \\ \frac{Qa_{j.m} * N_{j.m}}{N_{max\,j.m}} - Qa_{j.j} & ;\ N_{j.m} \geq N_{max} \end{cases} \quad \text{(Eq. 19)}$$

Water quality management options

$$N_{j.m} = \frac{(TDN_j/12)}{Qa_{j.m}} \quad \text{(Eq. 8)}$$

$$TDN_j = DIN_j + DON_j + \sum_{j2=j+1}^{n}(RS_{DIN.j2} * FE_{riv_{DIN.\,j2-j}} + RS_{DON.j2} * FE_{riv_{DON.\,j2-j}}) \quad \text{(Eq. 9)}$$

$$DIN_j = (RSdif_{DIN.j} + RSpnt_{DIN.j} + RSothers_{DIN.j}) * FE_{riv_{DIN.\,j}} \quad \text{(Eq. 10)}$$

$$DON_j = (RSdif_{DON.j} + RSpnt_{DON.j} + RSothers_{DON.j}) * FE_{riv_{DON.\,j}} \quad \text{(Eq. 11)}$$

$$RSpnt_{F.j} = \sum_{Opnt=1}^{8} Xpnt_{Opnt.\,j} * (1 - REpnt_{Opnt}) * FE_{pnt_{F,\,Opnt}} \quad \text{(Eq. 12)}$$

$$Rsdif_{F.j} = WSdiftotal_j - WSdifexport_j) * FE_{WS_{F.j}} \quad \text{(Eq. 13)}$$

$$WSdiftotal_j = \sum_{Odif=1}^{3} (Xdif_{Odif.j}) + WSdifothers_j \quad \text{(Eq. 14)}$$

$$X_{matotal.\,j} = Xdif_{Odif2.\,j} + Xdif_{Odif3.\,j} + Xpnt_{Opnt1.\,j} + Xpnt_{Opnt2.\,j} + Xpnt_{Opnt3.\,j} + Xpnt_{Opnt4.\,j} \quad \text{(Eq. 15)}$$

$$X_{hwtotal.\,j} = 2050WSdif_{hwland.\,j} + Xpnt_{Opnt5.\,j} + Xpnt_{Opnt6.\,j} + Xpnt_{Opnt7.\,j} + Xpnt_{Opnt8.\,j} \quad \text{(Eq. 16)}$$

Notations:

TC is the total annual cost of management options to mitigate water scarcity (billion $).
TC_{agr} is the total annual cost of management options in the agricultural sector (billion $).
TC_{dom} is the total annual cost of management options in the domestic sector (billion $).
TC_{STOR} is the total annual cost of water storage (billion $).
TC_{TRANS} is the total annual cost of water transport. TCN is the total annual cost of nitrogen sources (billion $).
$Da_{j.m}$ is the total agricultural water use in sub-basin j in month m (billion m^3).
Sa_{Oagr} is the share of water use that is saved by implementing management options Oagr (0-1).
$Xa_{j.Oagr}$ is the share of the agricultural area in sub-basin j where management options Oagr are implemented (0-1).
C_{Oagr} is the cost of water use for management options Oagr ($/$m^3$).
$Xd_{j.\ Odom}$ is the share of the total population in sub-basin j that implements management option Odom (0-1).
Sd_{Odom} is the domestic water saving from water efficient technology Odom (billion m^3/capita/year).
P_j is the total population in sub-basin j (number of individuals).
C_{Odom} is the cost of one unit of management options Odom (billion $).
$STORin_{j\ m}$ is the amount of water that goes into the storage in sub-basin j in month m (billion m^3).
$STORout_{j\ m}$ is the amount of water that is taken out of the storage in sub-basin j in month m (billion m^3).
C_{STOR} is the cost of storing water in reservoirs ($/$m^3$).
$TRANSin_{j\ .j2\ .m}$ is the amount of water that is transported from basin j to basin j2 in month m (billion m^3).
$TRANSout_{j\ .j2\ .m}$ is the amount of water that is transported to sub-basin j2 from basin j in month m (billion m^3).
$C_{TRANS.\ j.\ j2}$ is the cost of transporting water from sub-basin j to sub-basin j2 ($/ m^3).
$Qa_{j.m}$ is the total available water discharge in sub-basin j in month m (billion m^3).
$Qn_{j.m}$ is the natural river discharge in sub-basin j in month m (billion m^3).
$N_{j.m}$ is the nitrogen concentration in sub-basin j in month m(mg/l).
TDN_j is the total annual dissolved nitrogen at the outlet of sub-basin j (kton).
$N_{max_{j.m}}$ is the water quality standard for nitrogen concentration (mg/l). In this study, the water quality standard of nitrogen for thedomestic sector of 1.0 mg/l is used.
DIN_j is the total dissolved inorganic nitrogen at the outlet of sub-basin j (kton).
DON_j is the total dissolved organic nitrogen at the outlet of sub-basin j (kton).
$RS_{DIN.j2}$ is the total amounts of inorganic nitrogen input to the rivers in sub-basin j2 (kton).
$RS_{DON.j2}$ is the total amounts of organic nitrogen input to the rivers in sub-basin j2 (kton).
$FE_{riv_{DIN.\ j2-j}}$ is the export fractions of DIN from sub-basin j2 that are exported to the outlet of sub-basin j (0-1).
$FE_{riv_{DON.\ j2-j}}$ is the export fractions of DON from sub-basin j2 that are exported to the outlet of sub-basin j (0-1).$RSdif_{DIN.j}$ is the input of inorganic nitrogen to the river from diffuse sources in sub-basin j (kton).
$RSdif_{DON.j}$ is the input of organic nitrogen to the river from diffuse sources in sub-basin j (kton).
$RSpnt_{DIN.j}$ is the input of inorganic nitrogen to the river from point sources in sub-basin j (kton).
$RSpnt_{DON.j}$ is the input of organic nitrogen to the river from point sources in sub-basin j (kton). $RSothers_{DIN.j}$ is the input of inorganic nitrogen to the river from other sources in sub-basin j (kton).
$RSothers_{DON.j}$ is the input of organic nitrogen to the river from other sources in sub-basin j (kton).
$FE_{riv_{DIN.\ j}}$ is the export fractions of inorganic nitrogen to the outlet of sub-basin j (0-1).
$FE_{riv_{DON.\ j}}$ is the export fractions of organic nitrogen to the outlet of sub-basin j (0-1).
$Xdif_{Odif.\ j}$ is the amount of nitrogen applied to land with management option Odif in sub-basin j (kton).
$Xpnt_{Opnt.\ j}$ is the amount of nitrogen applied to land with management option Opnt in sub-basin j (kton).
$CFdif_{Odif}$ is the nitrogen content factor of management options Odif (kton/kton).
$CFpnt_{Opnt}$ is the nitrogen content factor of management options Opnt (kton/kton).
C_{Odif} is the cost of management options Odif ($/kton).
C_{Opnt} is the cost of management options Opnt ($/kton).
$Rsdif_{F.j}$ is the total amounts of nitrogen input to the river from diffuse sources as nutrient form F (DIN, DON) in sub-basin j (kton).
$RSpnt_{F.j}$ is the total amounts of nitrogen input to the river from point sources as nutrient form F (DIN, DON) in sub-basin j (kton).
$WSdiftotal_j$ is the total input of nitrogen to agricultural land in sub-basin j (kton).
$WSdifexport_j$ is the export of nitrogen from agricultural land by cropharvesting and grazing in sub-basin j (kton).
$WSdifothers_j$ is the nitrogen input to agricultural land from other diffuse sources in sub-basin j (kton). $REpnt_{Opnt}$ is the removal efficiency of nitrogen in the treatment of management option Opnt (0-1).
$FE_{WS_{F.j}}$ is the export fraction of nitrogen from land to rivers as diffuse sources in nutrient form F (DIN, DON) in sub-basin j (0-1).
$FE_{pnt_{F,\ Opnt}}$ is the export fraction of nitrogen from point sources as nutrient form F (DIN, DON) that enters the river after treatment of management option Opnt(0-1).
$X_{matotal.\ j}$ is the total projected amount of nitrogen in human waste in 2050 in sub-basin j (kton).

$\boldsymbol{X_{hwtotal.\ j}}$ is the total projected amount of nitrogen in human waste in 2050 in sub-basin j (kton).
$\mathbf{2050}\boldsymbol{WSdif_{hwland.\ j}}$ is the projected amount of nitrogen from human waste that stays on land in 2050 (kton).
$\boldsymbol{WSq_{j.m}}$ is the level of water scarcity in sub-basin j in month m (unitless).
$\boldsymbol{WSq_{max}}$ is water scarcity that is constrained.
$\boldsymbol{D_{j.m}}$ is water demand in sub-basin j in month m (billion m^3).
$\boldsymbol{TS_{j.m}}$ is the total water savings in sub-basin j in month m (billion m^3),
$\boldsymbol{TS_{j2.m}}$ is the total water savings in upstream sub-basin j2 in month m (billion m^3).
$\boldsymbol{dq_{j.m}}$ is the total amount of water needed for dilution to reach the water of sufficient quality in sub-basin j in month m (billion m^3).
$\boldsymbol{EFR_{j.m}}$ is the total amount of water for the environmental flow requirements (billion m^3), and it was calculated as 37% of the available water discharge.

Supplementary note 3. Water availability (VIC hydrological model)

The total available water quantity (Q) is defined in this study as the natural river discharge of the study area (billion m^3). Natural river discharge is first calculated on a grid scale and then upscaled to sub-basin and river basin scales. Natural river discharge is derived in m^3 per second at a 0.5° longitude by 0.5° latitude global scale grid for the years 1961-2099 from the global hydrological VIC (Variable Infiltration Capacity) model[4]. In this study, natural river discharges for both the RCP2.6 and RCP8.5 climate scenarios are calculated as the average of the five outputs from the VIC model forced with five different general circulation models [5]. This is done to reduce the uncertainties that occur within the different general circulation models. Natural river discharge for 2010 and 2050 is then calculated as a 30-year average to account for possible yearly extremes. For the year 2010 natural river discharge is calculated as the 30-year average of the years 1981-2010, and for 2050 as the 30-year average of the years 2036-2065.

For both 2010 and 2050 natural river discharge in the Pearl River Basin is derived from this global grid in GIS. A map with the outline of the Pearl River Basin is derived from the ddm30 river basin map [2]. The total annual natural river discharge in the cells of the Pearl River Basin is calculated by multiplying the natural river discharge values (in m^3/s) with the total amount of seconds in a year and is given in billion m^3.

The total modeled annual natural river discharge in 2010 is then adjusted with the observed total annual river discharge of the Pearl River in 2010. This is done to reduce the uncertainty in the globally modeled data from the VIC model in this regional assessment of the Pearl River Basin. The harmonization is done by multiplying the modeled total annual natural river discharge in every 0.5° x 0.5° cell in the Pearl River Basin, by the ratio between the total modeled annual natural river discharge in the Pearl River Basin in 2010 and the total observed annual natural river discharge in the Pearl River Basin in 2010. Total natural river discharge in a sub-basin is equal to the natural river discharge in the most downstream 0.5° x 0.5° cell of the sub-basin since this is the accumulated natural river discharge of all the cells in the sub-basin. For the whole Pearl River Basin the total natural river discharge is equal to the sum of the most downstream 0.5° x 0.5° cells of the Delta Zhujiang and the Dongjiang sub-basins.

For the sub-basins of the Pearl River Basin, the monthly natural river discharge is also calculated. This is done by multiplying the yearly total natural river discharge by monthly natural river discharge shares. The monthly shares are set the same for all the sub-basin of the Pearl River Basin and are calculated with observed monthly natural river discharge. Observed monthly natural river discharge data are taken from the Wuzhou river discharge measuring station, and are derived from the Global Runoff Data Centre [6]. Monthly discharges are calculated as the average discharge of a month over the years 1946-1986. Monthly shares are then calculated as the relative share of that month to the total discharge in a year. These monthly shares are then multiplied by the total yearly natural river discharge of the sub-basins for both 2010 and 2050.

Supplementary note 4. The Marginal Abatement Cost Curve (MACC).

The MACC is a tool for mitigation policy analysis that brings a wide range of information about mitigation management options and shows the potential of water scarcity abatement and the associated costs for different management options. The marginal abatement cost curve (MACC) was first used by McCarl and Schneider (2000)[7] to analyze mitigation policies in US agriculture. Subsequently, it has been used in different studies at global, regional and national levels [8-11]. The information provided by the MACC reveals which are the most effective policy interventions in order to facilitate the exchange between scientific studies and policy decision-making. The marginal cost of abatement can be calculated in various ways. Vermont and De Cara (2010) [12] divides MACCs into three main types based on the methodology used to derive the curves: (i) bottom up cost-engineering; (ii) micro-economic modelling, with exogenous prices; (iii) regional/sectoral supply-side equilibrium models.

In this study, the MACC was based on bottom-up cost engineering approach. In this approach, information on the one-off and recurring costs of a range of water management options and their effect on water saving and N pollution is collected. These data are used to calculate (a) the average annual cost of each option, (b) the amount of water scarcity reduced each year, and (c) the cost-efficiency, i.e. the cost per unit of water scarcity reduced. The MACC in Supplementary Figure 1 shows series of discrete bars that represents the rising costs and the % of water scarcity abatement from each water quantity and quality management option (e.g., low-pressure pipe irrigation, applying synthetic nitrogen fertilizer, recycling manure, and treating human waste). The width of each bar represents the reduction of water scarcity (%), while the height of the bar shows the cost-efficiency of the management options ($ Billion/1% of water scarcity abatement). The area under each bar is equal to the total cost of the management option. The different management options are ordered according to their cost-efficiency so that from left to right of thecurve the marginal abatement cost worsens as the accumulated abatement levels by management options increase and additional measures become more expensive. The abatementlevel of management options is related to water savings, water storage, water transport or reduced nitrogen loads. The marginal abatement cost of each management option is estimated by dividing the total cost of option implementation by the percentage of the water scarcity abatement of each option (Supplementary Figure 1).

Supplementary note 5. Water transport management option.

Water transport is a common water re-allocation practice in China with the implementation of China's South-North water transfer project [13]. The total cost of transporting water in this project is dependent on the total distance that the water is transferred and is given by Pohlner[14] as the price of thewater in the place where it is delivered. To get the cost of transporting water over any given distance, an exponential regression analysis is done on the costs and distances given in Supplementary Table 1 (R^2=0.9972). The resulting relationship between the cost of transporting water and distance is given by the equation:

$$C_{TRANS,j,j2}=0.0228\ e^{0.0032*Xj,j2}$$

Where Xj,j2 is the distance between sub-basin j and sub-basin j2 (km). The distances between the sub-basins of the Pearl River Basin are set as the distance in a straight line between the centers of the sub-basins (Supplementary Table 2). This is calculated with GIS with sub-basins of the ddm30 sub-basins map [2].

Supplementary Table 1. Cost of transporting water in China's South-North water transfer project fordifferent cities, and distance of the cities from the source region (Dengzhou).

Cities in China	Cost ($\$/m^3$)	Distance from source (km)
Dengzhou	0.02	0
Nanyang	0.03	55
Zhengzhou	0.06	270
Anyang	0.09	430
Shijiazhuang	0.16	635
Tianjin	0.35	850
Beijiang	0.38	890

The costs are the total cost of transporting water and are derived from [14]. The distances are calculated as the accumulated straight-line distance between the centers of the consecutive cities from Denghzou to Beijiang.

Supplementary Table 2. Transport distances between the different sub-basins of the Pearl RiverBasin (thousand km).

	Liujiang	Yujiang	Xijiang	Beijiang	Dongjiang	Delta Zhujiang
Liujiang	0	318	173	452	607	402
Yujiang	318	0	164	696	815	585
Xijiang	173	164	0	605	747	525
Beijiang	452	696	605	0	172	157
Dongjiang	607	815	747	172	0	229
Zhujiang Delta	402	585	525	157	229	0

The distance between two sub-basins represents the distance in a straight line between the centers of the sub-basins.

Supplementary Table 3: Effects and costs of water quantity and quality management options.

Management options	Waters saving (% or dm³)	N removal efficiency $REpnt_{Opnt}$	Costs (C_{Oagr}; C_{Odom}; C_{STOR}; C_{Odif}; C_{Opnt})
**Water quantity management options**			
**Agricultural sector $Xa_{j.Oagr}$**			
Low-pressure pipe irrigation	42%	-	\$0.017/ $m^3$of water
Canal irrigation	30%	-	\$0.039/m^3 of water
Sprinkler irrigation	39%	-	\$-0.016/m^3 of water[b]
**Domestic sector $Xd_{j.Odom}$**			
Water-efficient bathroom faucet	2.2[a]	-	\$15/faucet unit
Water-efficient kitchen faucet	10.6[a]	-	\$15/faucet unit
Water efficient showerhead	18.4[a]	-	\$100/showerhead
Water efficient toilet	6.17[a]	-	\$420/toilet
Water-efficient washing machine	3.4[a]	-	\$670/washing machine
Water efficient dishwasher	1.3[a]	-	\$500/dishwasher
**Water transfers $TRANSin_{j.j2.m}$; $STOR_{j.m}$**			
Water transport	-	-	0.02-0.38
Water storage	-	-	\$0.72/m^3 of water[c]
**Water quality management options**			
**Diffuse sources $Xdif_{Odif.j}$**			
Apply synthetic nitrogen fertilizer	-	-	\$350/ton of N
Recycle manure as slurry on land	-	-	\$18/ton of animal manure
Recycle manure as solid on land	-	-	\$23/ton of animal manure
**Point sources $Xpnt_{Opnt.j}$**			
Treat manure with primary technologies	-	10%	\$5/ton of animal manure[d]
Treat manure with secondary technologies	-	60%	\$7/ton of animal manure[d]
Treat manure with tertiary technologies	-	90%	\$12/ton of animal manure[d]
Discharge untreated manure to rivers	-	0%	\$0/ton of human waste[d]
Treat human waste with primary technologies	-	23%	\$1.09/ton of human waste[d]
Treat human waste with secondary technologies	-	41%	\$1.17/ton of human waste[d]
Treat human waste with tertiary technologies	-	72%	\$1.56/ton of human waste[d]
Discharge untreated human waste to rivers	-	0%	\$0/ton of human waste[d]

C_{Oagr}; C_{Odom}; C_{STOR}; C_{Odif}; C_{Opnt} : costs of water use for management options. $Xa_{j.Oagr}$: the share of the agricultural area in sub-basin j where agricultural management options are implemented. $Xd_{j.Odom}$: the share of the population that implements domestic management options. $TRANSin_{j.j2.m}$: the total amount ofwater that is transported from sub-basin j to sub-basin j2 in month m. $STOR_{j.m}$: is equal to the quantity of water that is stored in reservoirs. $Xdif_{Odif.j}$; $Xpnt_{Opnt.\,j}$: are respectively the amount of nitrogen applied to land with diffuse and point management options (Supplementary note 3).

[a] dm³ of water per day per capita

[b] For canal irrigation the cost-effectiveness is negative at \$-0.016 per m³ of water, which means that the cost savings of canal irrigation are bigger than the cost of implementing this technology [15].

[c] The cost of storing water is given by [16] at €0.53/m³. To transfer euros to dollars, the euro/dollar exchange rate was used at 1.36.

[d] Total amount of animal and human waste that is treated with primary, secondary or tertiary technologies.

Supplementary Table 4. Data sources and their temporal and spatial variability. The meaning of all variables is provided in Supplementary Box 1 and Supplementary Note 3.

Data / parameter	Unit	Source	Temporal aggregation	Spatial aggregation
**For water quantity**				
$Qn_{j.m}$	billion m^3	Van vielt et al.[4]	m: month	j: sub-basin
$STORin_{j.m}$	billion m^3	Brunner et al.[17]	m: month	j: sub-basin
$STORout_{j.m}$	billion m^3	Brunner et al.[17]	m: month	j: sub-basin
$TRANSin_{j.j2.m}$	billion m^3	Rogers et al.[13]	m: month	j, j2: sub-basins
$TRANSiout_{j.j2.m}$	billion m^3	Rogers et al.[13]	m: month	j, j2: sub-basins
$D_{j.m}$	billion m^3	Wanders et al.[18]	m: month	j: sub-basin
$Da_{j.m}$	billion m^3	Wanders et al.[18]	m: month	j: sub-basin
$Dd_{j.m}$	billion m^3	Wanders et al.[18]	m: month	j: sub-basin
$Di_{j.m}$	billion m^3	Wanders et al.[18]	m: month	j: sub-basin
P_j	People	Jones and O'Neill[19]		j: sub-basin
**Water savings for water quantity management options (Agriculture options: Oagr; Domestic options Odom)**				
Sa_{Oagr}	0 – 1	Blanke et al.[20]		
Sa_{Odom}	billion m^3/capita	Rasoulkhani et al.[21]		
**For water quality (MARINA-Nutrients model, version 2.0)**				
$WSdiftotal_j$	Kton	Wang et al.[1]		j: sub-basin
$WSdifexport_j$	Kton	Wang et al.[1]		j: sub-basin
$WSdifothers_j$	Kton	Wang et al.[1]		j: sub-basin
$2050WSdif_{hwland.j}$	Kton	Wang et al.[1]		j: sub-basin
$X_{matotal.j}$	Kton	Wang et al.[1]		j: sub-basin
$X_{hwtotal.j}$	Kton	Wang et al.[1]		j: sub-basin
$RSothers_{DIN.j}$	Kton	Wang et al.[1]		j: sub-basin
$RSothers_{DON.j}$	Kton	Wang et al.[1]		j: sub-basin
**Retention fractions of DIN and DON in downstream sub-basins**				
$FE_{riv.DIN.j}$	0 – 1	Wang et al.[1]		j: sub-basin
$FE_{riv.DON.j}$	0 – 1	Wang et al.[1]		j: sub-basin
**Removal efficiencies and retention fraction of water quality management options**				
$REpnt_{Opnt}$	Kton	Strokal et al.[3]		
$FEpnt_{F.Opnt}$	0 – 1	Wang et al.[1]		
$CFdif_{Odif}$	kton/kton	Wang et al.[1]		
$CFpnt_{Opnt}$	kton/kton	Wang et al.[1]		
**For water scarcity**				
$EFR_{j.m}$	billion m^3	Pastor et al.[22]	m: month	j: sub-basin
N_{max}	mg/l	MEE[23]		
**Costs of water quantity and quality management options**				
C_{Oagr}	\$/m^3	Blanke et al.[20]		
C_{Odom}	\$/unit	Rasoulkhani et al.[21]		
C_{STOR}	\$/m^3	Grygoruk et al.[16]		
$C_{TRANSj.j2}$	\$/m^3	Pohlner[14]		j, j2: sub-basins
C_{Odif}	\$/ton	Strokal et al.[3]		
C_{Opnt}	\$/ton	Strokal et al.[3]		

Supplementary Table 5. Set-up of a sensitivity analysis for costs of water quantity and quality management options**..**

Options	Costs			
	**Unit**	**Base value in the original model run**	**50% increased cost relative to the base value**	**100% increased cost relative to the base value**
Discharge untreated human waste to rivers	$/ton of human waste [a]	0	0	
Treat human waste with secondary technologies	$/ton of animal manure [a]	7	10.5	14
Treat human waste with tertiary technologies	$/ton of human waste [a]	1.56	2.34	3.12
Recycle manure as slurry on land	$/ton of animal manure	18	27	36
Recycle manure as solid on land	$/ton of animal manure	23	34.5	46
Apply synthetic nitrogen fertilizer on land	$/ton of synthetic nitrogen fertilizer	350	525	700
Low pressure pipe irrigation	$/m^3 of water	0.017	As the base value	As the base value
Water transport	(see Supplementary Note 2)	0.02-0.38	As the base value	As the base value
Water storage	$/m^3 of water	0.72	As the base value	As the base value
Water saving kitchen faucet	$/faucet	15	As the base value	As the base value
Water saving showerhead	$/showerhead	100	As the base value	As the base value
Water saving bathroom faucet	$/faucet	15	As the base value	As the base value
Water saving washing machine	$/washing machine	670	As the base value	As the base value
Water saving toilet	$/toilet	420	As the base value	As the base value
Water saving dishwasher	$/dishwasher	500	As the base value	As the base value

The grey-highlighted management options reflect water quality options, with base cost values rising by +50% and +100%, while the base cost values of water quantity options are kept constant.

Supplementary Table 6. Sensitivity analysis results under the SSP-RCP scenarios, water scarcity level, and increased cost of water quality management options.

Low Water Scarcity Level (0.1)												
**Options**	**SSP2-RCP2.6**				**SSP2-RCP8.5**				**SSP5 -RCP8.5**			
	**WS abat. (%)**	**CE**	**CE (+50%)**	**CE (+100%)**	**WS abat. (%)**	**CE**	**CE (+50%)**	**CE (+100%)**	**WS abat. (%)**	**CE**	**CE (+50%)**	**CE (+100%)**
Treat human waste with tertiary technologies	25	0.0000014	0.0000021	0.000003	13	0.000003	0.000004	0.000005	14	0.000002	0.000004	0.000005
Recycle manure as slurry on land	2	0.00013	0.00019	0.00026	9	0.00003	0.00004	0.00006	10	0.00002	0.00004	0.00005
Apply synthetic nitrogen fertilizer on land	23	0.01	0.02	0.02	19	0.01	0.02	0.03	14	0.02	0.04	0.05
Low pressure pipe irrigation	3	0.09	0.09	0.09	3	0.09	0.09	0.09	3	0.09	0.09	0.09
Water transport	14	0.20	0.20	0.20	14	0.05	0.05	0.05	14	0.04	0.04	0.04
Water storage	34	5	5	5	43	3	3	3	44	3	3	3
Water saving kitchen faucet	0.11	15	15	15	0.11	15	15	15	0.12	14	14	14
Water saving showerhead	0.20	57	57	57	0.20	57	57	57	0.20	54	54	54
Water saving bathroom faucet	0.02	72	72	72	0.02	36	36	36	0.02	68	68	68
Water saving washing machine	0.14	267	267	267	0.14	23	23	23	0.15	427	427	427
**Medium Water Scarcity Level (0.2)**												
Discharge untreated human waste to rivers	-	-	-	-	-	-	-	-	0.43	0	0	0
Treat human waste with secondary technologies	-	-	-	-	-	-	-	-	12	4.38E-09	6.57E-09	8.76E-09
Treat human waste with tertiary technologies	24	0.0000014	0.0000016	0.0000022	12	0.000003	0.000004	0.000006	11	0.000003	0.000004	0.000005
Recycle manure as slurry on land	2	0.0001	0.0002	0.0003	8	0.00003	0.00005	0.00006	7	0.00003	0.00005	0.00007
Apply synthetic nitrogen fertilizer on land	21	0.012	0.018	0.023	17	0.01	0.02	0.03	11	0.03	0.05	0.06
Low pressure pipe irrigation	2	0.10	0.10	0.10	2	0.10	0.10	0.10	2	0.12	0.12	0.12
Water transport	19	0.22	0.22	0.22	8	0.17	0.17	0.17	9	0.22	0.22	0.22
Water storage	31	6	6	6	52	4	4	4	47	5	5	5
Water saving kitchen faucet	0.10	16	16	16	0.10	16	16	16	0.09	19	19	19
Water saving showerhead	0.18	62	62	62	0.18	62	62	62	0.15	73	73	73
Water saving bathroom faucet	0.02	78	78	78	0.02	79	79	79	0.02	93	93	93
Water saving washing machine	0.26	291	291	291	0.26	292	292	292	0.21	344	344	344
Water saving toilet	0.06	771	771	771	0.06	773	773	773	0.05	913	913	913

Water saving dishwasher	0.01	4311	4311	4311	0.01	4323	4323	4323	0.01	5109	5109	5109
**High Water Scarcity Level (0.4)**												
**Options**	**SSP2-RCP2.6**				**SSP2-RCP8.5**				**SSP5 -RCP8.5**			
	**WS abat. (%)**	**CE**	**CE (+50%)**	**CE (+100%)**	**WS abat. (%)**	**CE**	**CE (+50%)**	**CE (+100%)**	**WS abat. (%)**	**CE**	**CE (+50%)**	**CE (+100%)**
Recycle manure as solid on land	23	3.09E-08	4.63E-08	6.17E-08	-	-	-	-	-	-	-	-
Treat human waste with tertiary technologies	19	0.000002	0.000003	0.000004	12	0.000003	0.000004	0.000005	14	0.000003	0.000004	0.000006
Recycle manure as slurry on land	2	0.00015	0.00023	0.00031	8	0.00003	0.00004	0.00006	9	0.00003	0.00004	0.00005
Apply synthetic nitrogen fertilizer on land	17	0.01	0.02	0.03	18	0.014	0.020	0.027	13	0.019	0.039	0.051
Low pressure pipe irrigation	2	0.12	0.12	0.12	3	0.09	0.09	0.09	3	0.09	0.09	0.09
Water transport	11	0.26	0.26	0.26	7	0.16	0.16	0.16	6	0.16	0.16	0.16
Water storage	26	6	6	6	51	4	4	4	55	4	4	4
Water saving kitchen faucet	0.09	19	19	19	0.11	15	15	15	0.11	15	15	15
Water saving showerhead	0.15	75	75	75	0.19	59	59	59	0.19	58	58	58
Water saving bathroom faucet	0.02	95	95	95	0.02	75	75	75	0.02	74	74	74
Water saving washing machine	0.21	351	351	351	0.27	278	278	278	0.27	273	273	273
Water saving toilet	0.05	930	930	930	0.06	736	736	736	0.06	723	723	723
Water saving dishwasher	0.01	5202	5202	5202	0.01	4117	4117	4117	0.01	4045	4045	4045

The grey-highlighted management options reflect water quality options. **WS abat.** represents the potential abatement of water scarcity (%), and **CE** is the cost-efficiency of management options for different values of the cost of water quality options (base value; +50%; +100% of the unit cost; see supplementary table 4) under the SSP-RCP scenarios (SSP2-RCP2.6; SSP2-RCP8.5; SSP5-RCP8.5) and water scarcity levels (low, medium, high), ($ billion/% of water scarcity abatement). RCP stands for Representative Concentration Pathway. SSP stands for Shared Socioeconomic Pathway.

Depending on the scenario and water scarcity level, different combinations of management options could be selected by the optimization model.

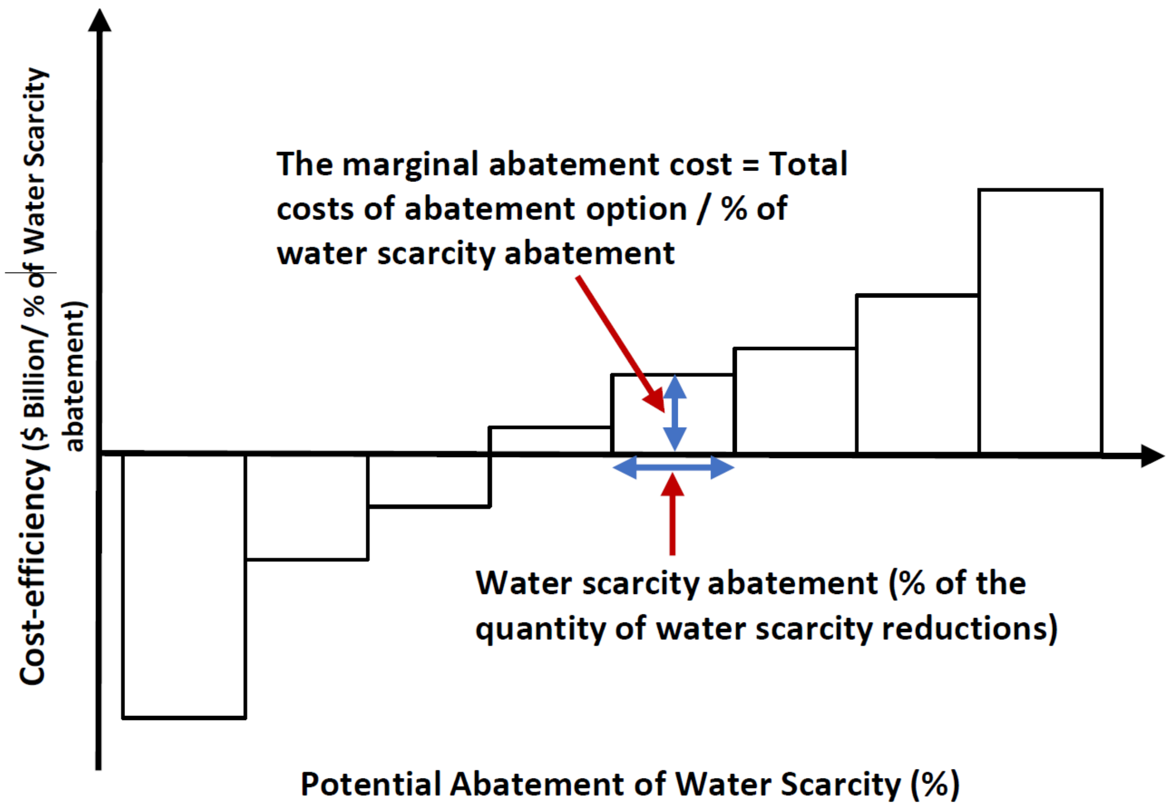

Supplementary Figure 1. Marginal abatement cost Curve (MACC).

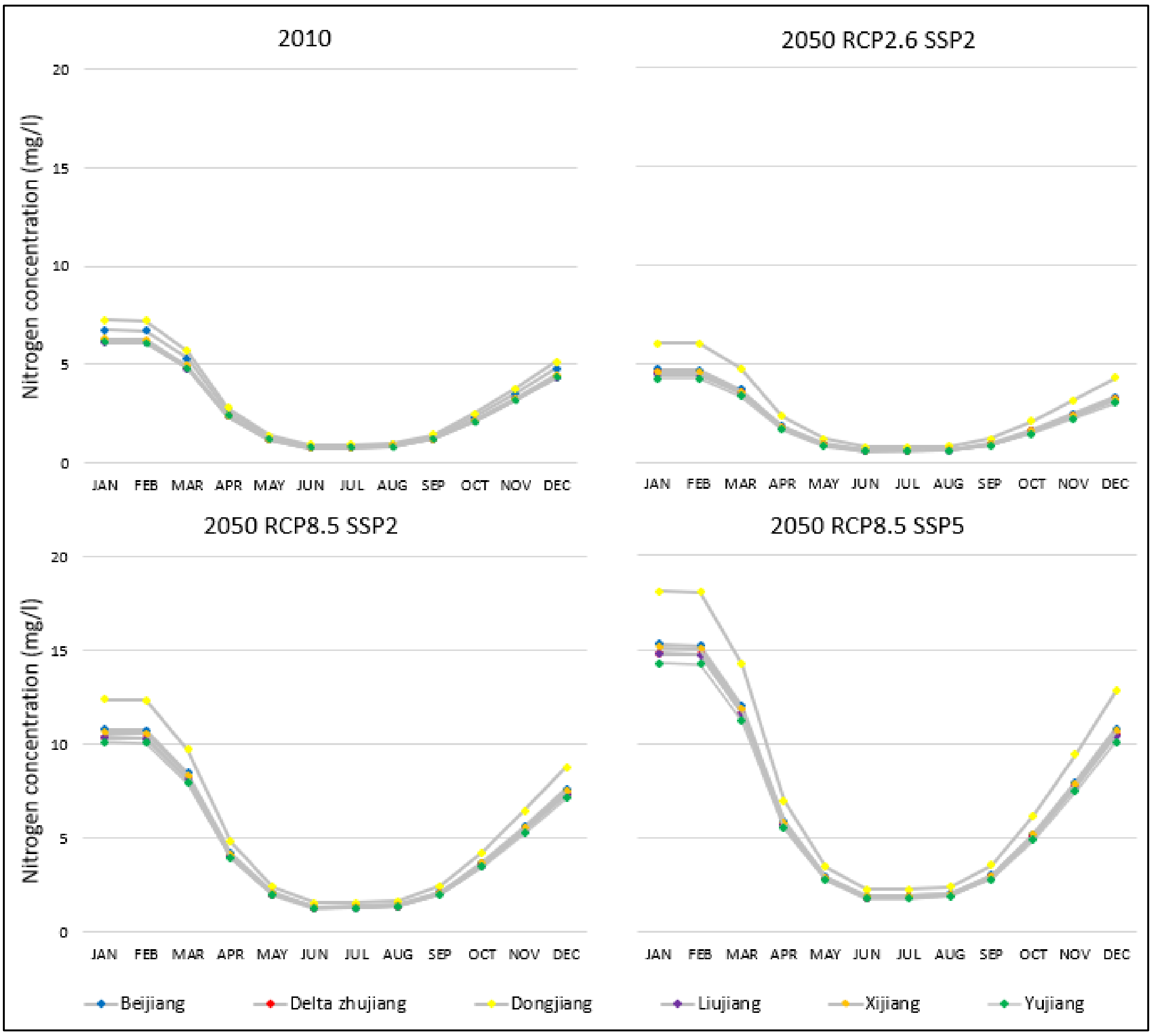

Supplementary Figure 2. Monthly nitrogen concentrations in the rivers of the Pearl River sub-basins in 2010 and in 2050 for the RCP2.6-SSP2, RCP8.5-SSP2, and RCP8.5-SSP5 scenarios (mg/l). RCP is short for Representative Concentration Pathway, and SSP is short for Shared Socio-economic Pathway, see section 2.1.1 for scenario descriptions. Source: Calculated with nitrogen data from the MARINA-Nutrients model (version 2.0) (Wang et al., 2020) combined with discharge data from the VIC model (Van Vliet et al., 2016).

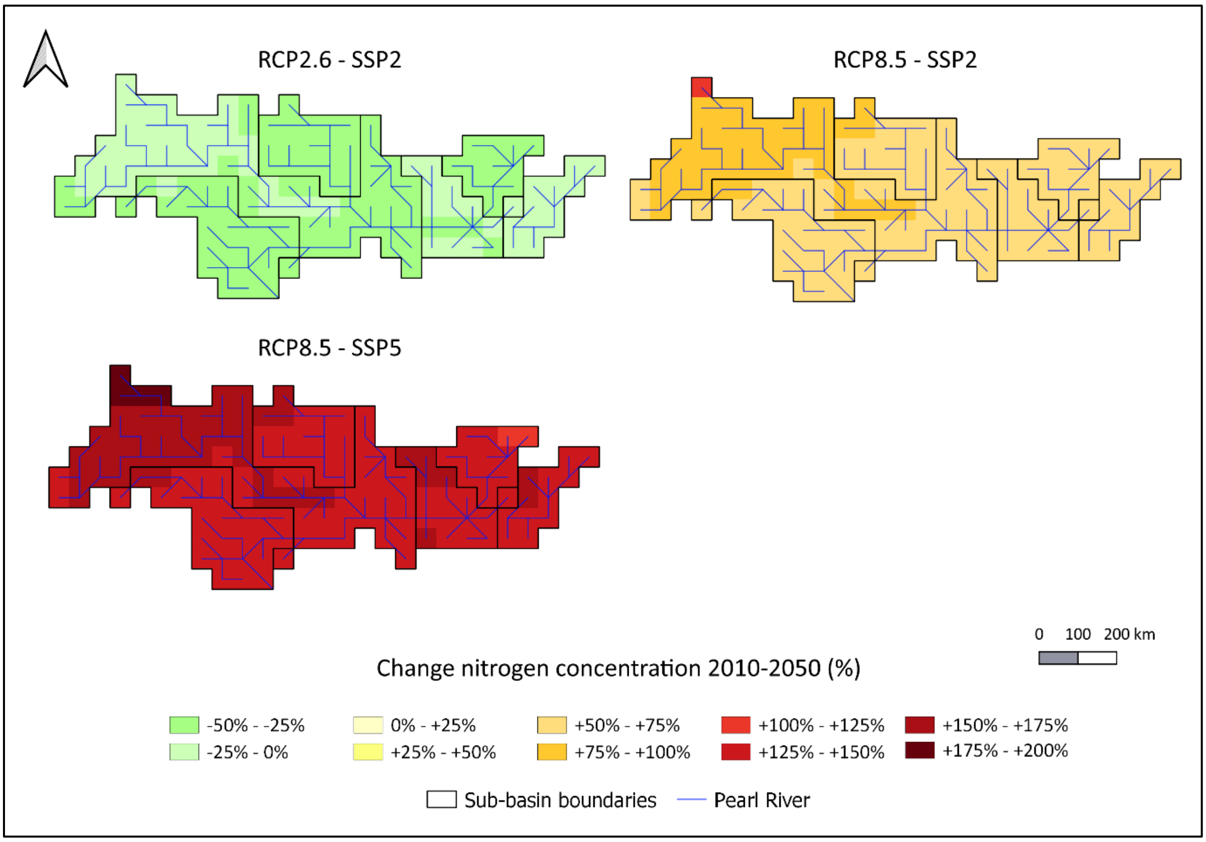

Supplementary Figure 3. Changes in nitrogen concentration in the rivers of the Pearl River Basin between 2010 and 2050 for the RCP2.6-SSP2, RCP8.5-SSP2, and RCP8.5-SSP5 scenarios at 0.5° x 0.5° grid (%). These data were aggregated from sub-basins (Supplementary Figure 2) to grids using the description in Supplementary Note 2.

Supplementary references

1. Wang, M. R., Kroeze, C., Strokal, M., van Vliet, M. T. H. & Ma, L. Global Change Can Make Coastal Eutrophication Control in China More Difficult. *Earth's futur.* **8**, 19, doi:10.1029/2019ef001280 (2020).
2. Doll, P. & Lehner, B. Validation of a new global 30-min drainage direction map. *J. Hydrol.* **258**, 214-231, doi:10.1016/s0022-1694(01)00565-0 (2002).
3. Strokal, M. *et al.* Cost-effective management of coastal eutrophication: A case study for the yangtze river basin. *Resour. Conserv. Recycl.* **154**, 11, doi:10.1016/j.resconrec.2019.104635 (2021).
4. van Vliet, M. T. H. *et al.* Multi-model assessment of global hydropower and cooling water discharge potential under climate change. *Glob. Environ. Change-Human Policy Dimens.* **40**, 156-170, doi:10.1016/j.gloenvcha.2016.07.007 (2016).
5. van Vliet, M. T. H. *et al.* Multi-model assessment of global hydropower and cooling water discharge potential under climate change. *Glob. Environ. Change-Human Policy Dimens.* **40**, 156-170, doi:10.1016/j.gloenvcha.2016.07.007 (2016).
6. (GRDC)., G. R. D. C. In-situ river discharge data for your research project. Federal Institute of Hydrology (BfG). Retrieved from https://www.bafg.de/GRDC/EN/02_srvcs/21_tmsrs/riverdischarge_node.html. (2020).
7. McCarl B.A. & U.A., S. U.S. Agriculture’s Role in a Greenhouse Gas Emission Mitigation World: An Economic Perspective. . *Appl. Econ. Perspect Policy. 2000;22:134–159. doi: 10.1111/1058-7195.t01-1-00011.* (2000).
8. Albiac, J., Kahil, T., Notivol, E. & Calvo, E. Agriculture and climate change: Potential for mitigation in Spain. *Sci. Total Environ.* **592**, 495-502, doi:10.1016/j.scitotenv.2017.03.110 (2017).
9. MacLeod, M. *et al.* Developing greenhouse gas marginal abatement cost curves for agricultural emissions from crops and soils in the UK. *Agric. Syst.* **103**, 198-209, doi:10.1016/j.agsy.2010.01.002 (2010).
10. Pellerin, S. *et al.* Identifying cost-competitive greenhouse gas mitigation potential of French agriculture. *Environ. Sci. Policy* **77**, 130-139, doi:10.1016/j.envsci.2017.08.003 (2017).
11. Wang, W. *et al.* Greenhouse gas mitigation in Chinese agriculture: Distinguishing technical and economic potentials. *Glob. Environ. Change-Human Policy Dimens.* **26**, 53-62, doi:10.1016/j.gloenvcha.2014.03.008 (2014).
12. Vermont, B. & De Cara, S. How costly is mitigation of non-CO₂ greenhouse gas emissions from agriculture? A meta-analysis. *Ecol. Econ.* **69**, 1373-1386, doi:10.1016/j.ecolecon.2010.02.020 (2010).
13. Rogers, S. *et al.* An integrated assessment of China's South-North Water Transfer Project. *Geogr. Res.* **58**, 15, doi:10.1111/1745-5871.12361 (2020).
14. Pohlner, H. Institutional change and the political economy of water megaprojects: China's south-north water transfer. *Glob. Environ. Change-Human Policy Dimens.* **38**, 205-216, doi:10.1016/j.gloenvcha.2016.03.015 (2016).
15. Zou, X. X. *et al.* Cost-effectiveness analysis of water-saving irrigation technologies based on climate change response: A case study of China. *Agric. Water Manag.* **129**, 9-20, doi:10.1016/j.agwat.2013.07.004 (2013).
16. Grygoruk, M., Mirosław-Światek, D., Chrzanowska, W. & Ignar, S. How Much for Water? Economic Assessment and Mapping of Floodplain Water Storage as a Catchment-Scale Ecosystem Service of Wetlands.Water (Switzerland), 5(4), 1760–1779. https://doi.org/10.3390/w5041760. (2013).
17. Brunner, M. I. *et al.* Present and future water scarcity in Switzerland: Potential for alleviation through reservoirs and lakes. *Sci. Total Environ.* **666**, 1033-1047, doi:10.1016/j.scitotenv.2019.02.169 (2019).
18. Wanders, N., Wada, Y., Van Beek, R. & Bierkens, M. Original data for study on Human water consumption intensifies hydrological drought worldwide. Retrieved

from https://doi.org/10.34894/3U3FG1. (2018).
19 Jones, B. & O'Neill, B. C. Spatially explicit global population scenarios consistent with the Shared Socioeconomic Pathways. *Environ. Res. Lett.* **11**, 10, doi:10.1088/1748-9326/11/8/084003 (2016).
20 Blanke, A., Rozelle, S., Lohmar, B., Wang, J. X. & Huang, J. K. Water saving technology and saving water in China. *Agric. Water Manag.* **87**, 139-150, doi:10.1016/j.agwat.2006.06.025 (2007).
21 Rasoulkhani, K., Logasa, B., Reyes, M. P. & Mostafavi, A. Understanding Fundamental Phenomena Affecting the Water Conservation Technology Adoption of Residential Consumers Using Agent-Based Modeling. *Water* **10**, 24, doi:10.3390/w10080993 (2018).
22 Pastor, A. V., Ludwig, F., Biemans, H., Hoff, H. & Kabat, P. Accounting for environmental flow requirements in global water assessments. *Hydrol. Earth Syst. Sci.* **18**, 5041-5059, doi:10.5194/hess-18-5041-2014 (2014).
23 MEE. Environmental Quality Standards for Surface Water (GB3838-2002). (2002).
