# Supplementary material for: Water quality management could halve future water scarcity cost-effectively in the Pearl River Basin
Source: Nat Commun. 2024 Jul 6;15:5669. doi: 10.1038/s41467-024-49929-z (PMC11227540; doi:10.1038/s41467-024-49929-z)
Supplement: Supplementary file 2 — Peer Review File [file 41467_2024_49929_MOESM2_ESM.pdf]

nature portfolio

Peer Review File

Water quality management could halve future water scarcity cost-effectively in the Pearl River Basin

Open Access This file is licensed under a Creative Commons Attribution 4.0 International License, which permits use, sharing, adaptation, distribution and reproduction in any medium or format, as long as you give appropriate credit to the original author(s) and the source, provide a link to the Creative Commons license, and indicate if changes were made. In the cases where the authors are anonymous, such as is the case for the reports of anonymous peer reviewers, author attribution should be to 'Anonymous Referee' followed by a clear attribution to the source work. The images or other third party material in this file are included in the article's Creative Commons license, unless indicated otherwise in a credit line to the material. If material is not included in the article's Creative Commons license and your intended use is not permitted by statutory regulation or exceeds the permitted use, you will need to obtain permission directly from the copyright holder. To view a copy of this license, visit http://creativecommons.org/licenses/by/4.0/.

REVIEWER COMMENTS

Reviewer #1 (Remarks to the Author):

The manuscript "Water quality management could cost-effectively halve future water scarcity" by Baccour et al. combines the Model to Assess River Inputs of pollutaNts to seAs (MARINA-Nutrients model) and a cost-optimization procedure to explore effective solutions to reduce future water scarcity in the Pearl River Basin. Overall, the idea of including water quality conditions into water scarcity assessment with distributed watershed model is novel and offers potential benefits for future water management. While my major concern is the uncertainties within this study and the clarity of the methodologies utilized.
On the whole, I recommend a major revision considering the following comments.
My major comments:
1. The methodology section requires further clarity and detail. The unique aspect of this study is its consideration of water quality scarcity, making the results of the MARINA model central to this approach. My understanding is that the authors directly adopted the model results from a previous study (Wang et al., 2020), and incorporated them into this methodology. Please clarify if this is the case. As the authors aim to provide a framework for future assessments of water scarcity in other basins, a clear articulation of the methodology and data use is essential. Details such as databases used, temporal and spatial scales, could be presented in a table for greater clarity regarding the construction process and foundation of the method.

2. Uncertainty analysis or sensitivity analysis is crucial for the research of model simulation and future scenario forecasting. The manuscript or supplementary material should present some key calibration results from the MARINA model. I understand these results are adopted from a previous study, since the including of water quality based on MARINA model is the novelty of this study, it is important to discuss the uncertainty, not only the inherent uncertainty of the MARINA model. The authors should explore and discuss how these uncertainties might propagate into the final results of this method, and how they might impact predictions for the future.

3. The manuscript refers to the use of the General Algebraic Modeling System (GAMS) for optimization. Can the optimization results be processed in conjunction with uncertainties from the MARINA model?

4. The nitrogen legacy effect is crucial, which has been increasingly recognized and emphasized in global watershed studies. Can you discuss some potential implications of the legacy effect and its future impact on water quality scarcity.
5. In Supplementary note 2, the MARINA-Nutrients model is described to calculate N concentrations annually, and the amount of dissolved nitrogen is assumed to be equal across all months (Line 57-60, Supplementary note). Such an assumption may introduce uncertainty when comparing and discussing monthly results (Fig. 2). The discussion on seasonal variations of water scarcity levels should consider the uncertainty introduced by this assumption.

Reviewer #2 (Remarks to the Author):

Water scarcity is a global challenging, and has been increasingly concerned by both water managers and researchers during the past decade. Therefore, I believe the topic of the manuscript is interesting, and well in the scope of NC. However, I am not well convinced. Detailed comments were given as follows.
1) Title: The title is interesting. However, I am not well convinced. The study area of this study is the Pearl River Basin, a large watershed in south China. It is a regional study, rather than national or global study. Therefore, the conclusion (title) may not be true for other watersheds. In particularly, in

north China, the water resources are not as rich as the study area (Pearl River Basin), water quality management options would be much more costly.
2) Abstract: authors claimed model coupling (water quality model with a cost-optimization procedure) as novel modeling approach. I am not fully agreed. Coupling a process-based model with a cost-optimization procedure has been published before, e.g., https://doi.org/10.1016/j.spc.2022.01.031.
3) Introduction: "To our knowledge, there is no study available using the cost-efficiency of management options to reduce water scarcity". I am not fully agreed with this statement. This issue has been investigated in previous publications, e.g., https://doi.org/10.3390/w14193056. Therefore, I suggest authors to better highlight the contribution of this study.
4) Line 261: This paragraph discussed the model uncertainties from model structure. However, the data from climate and socioeconomic scenarios, and the costs of water quantity and quality management options (Supplementary Table 3) may be another uncertainty sources.
5) Line 275: The model included only N as a water quality parameter. Why not add P as another water quality parameter. Compared to N concentration, P concentration is more highly concerned by water managers due to its highly related to lake eutrophication and harmful algal blooms.
6) Cost calculation: I guess the investigated results are very sensitive to the cost calculation parameters (i.e., Supplementary Table 3). I fully understand it is extremely challenging to determine these parameter values. However, I believe considerable efforts are needed to convince the use of these values. For example, the cost of water transport is 0.02-0.38. However, I am not sure if it is able to carry our water transfer projects in these areas. I do not have too much experiences at the study area (Pearl River Basin), and was not able to evaluate the reasonability of these parameters. Moreover, references for these values would be helpful.
7) Model validation: The authors stated that "The model has been validated and evaluated by Wang, et al. 40". i) I think some brief description on the validation results (validated data, model fit, etc.) are needed. ii) I have a quick scan on the reference. To be honest, I am not very satisfied on the validated results of this study. In particularly, the reference compared the estimated nutrient load (annual) with measurement. This would achieve a high model fit compared to the comparison based on nutrient concentration.

Reviewer #3 (Remarks to the Author):

This paper integrates established approaches to measuring water quantity and water quality to develop a single, integrated quantity/quality characterization of water scarcity in China's Pearl River Basin. The authors project both water quality (using nitrogen concentration as the sole parameter of interest) and water quantity (river discharge) at the 0.5-km2 grid cell level to 2050 under three different future climate/socioeconomic conditions. They then estimate the costs and benefits (in terms of reduced water scarcity) of various mitigation approaches, including agricultural water quality and irrigation efficiency measures, urban residential water efficiency investments, and water storage and transport infrastructure investments. They conclude that measures to reduce N inputs from the agricultural sector (the "water quality" options) are more cost-effective approaches to reducing the future occurrence of water scarcity conditions in the Pearl River basin.

This is interesting work, and it represents a contribution relative to the prior literature. I have some more specific comments for the authors, which I hope will be useful as they continue to revise their work.

1. The paper does not provide enough description of the water quality criterion used to determine whether each part of the Basin has water of sufficient ambient quality for its designated use. Lines 122-124 suggest that areas with an average concentration above 1.0mg/l of N "do not meet the N quality standard...for all sectors." In the United States, the maximum contaminant level (MCL) for nitrite in finished drinking water is 1mg/l. The MCL for nitrate in finished drinking water is 10mg/l. The WHO standard for nitrate in finished drinking water is even higher (50mg/l as NO3, or 11.3mg/l as NO3-N). The paper does not provide enough background on water quality in the Basin to help the

reader understand whether nitrate or nitrite is the greater concern, and how they chose the 1mg/l standard for ambient concentrations in the river. This seems very stringent - can the authors explain this better, and if their threshold is too low, how do the results change if it is raised?

2. I realize that the water scarcity measure, itself, is the subject of a separate, published paper, and it is partially explained in the supplemental information. However, I found it jarring to read the results section, which simply applies the water scarcity measure without explaining to the reader what it is - how it is calculated, that it is unitless, and how we should interpret it. Relatedly, please define the terms in the denominator of the water scarcity measure equation (1) in the supplemental information.

3. Figure 2 is important to the paper, but I found it very difficult to read. Here are some suggestions:
(a) Either drop 2(a), or explain how to interpret this sub-figure in both the figure notes and in the text of the paper. I found that portion of Fig. 2 totally un-interpretable.
(b) I found it confusing that N is measured in red and water quantity in blue in 2(b), but the opposite color scheme is applied in 2(c).
(c) In 2(b) and (c), it seems clear that there is no meaningful change in river discharge, at least at the basin scale, under the three different RCP/SSP scenarios. This bears mentioning in the paper. Given this fact, it is not terribly surprising that addressing the water quality problems (which do change substantially under the three scenarios) are really the only cost-effective path to mitigating scarcity.

4. Figure 3 is also important to the paper. But given the description in Supplementary Note 5 of how the MACC were calculated (dividing TC by the % of water scarcity abatement achieved with each option), these are not marginal cost curves. They are average cost curves. Marginal cost curves would not include fixed costs.

5. The discussion section is too long and repetitive of text that appears earlier in the paper. It also introduces some new results (in lines 223-232, for example, describing specific agricultural water quality management options). I suggest moving that discussion to the results section, and otherwise substantially shortening the discussion.

3.

Response to comments on the revised perspective paper "Water quality management could cost-effectively halve future water scarcity". It was prepared by Safa Baccour, Gerwin Goelema, Taher Kahil, Jose Albiac, Michelle T.H. van Vliet, Xueqin Zhu, Maryna Strokal.

Foremost, we would like to thank the three reviewers for the valuable comments that have helped us to improve substantially the quality of the paper. We also thank the editor for giving us another chance to improve the response letter and perform extra model runs to better understand the influence of uncertainties on the main conclusions of our manuscript. We believe that our manuscript has been greatly improved. We have responded to all comments as below. The revisions we have made are highlighted in the revised manuscript and supplementary information in red. The revised texts are also provided in our responses below, where appropriate. Please note that we updated the affiliations of some of the authors (indicated in red in the revised manuscript).

Reviewer #1:

Comment 1: *The manuscript "Water quality management could cost-effectively halve future water scarcity" by Baccour et al. combines the Model to Assess River Inputs of pollutaNts to seAs (MARINA-Nutrients model) and a cost-optimization procedure to explore effective solutions to reduce future water scarcity in the Pearl River Basin. Overall, the idea of including water quality conditions into water scarcity assessment with distributed watershed model is novel and offers potential benefits for future water management. While my major concern is the uncertainties within this study and the clarity of the methodologies utilized. On the whole, I recommend a major revision considering the following comments.*

Our response: We thank the reviewer for the positive evaluation of our manuscript and the thoughtful comments provided. In our responses below, we explain how we addressed the comments of the reviewer and incorporated them into the revised manuscript.

Comment 2: *My major comments:*
1. The methodology section requires further clarity and detail. The unique aspect of this study is its consideration of water quality scarcity, making the results of the MARINA model central to this approach. My understanding is that the authors directly adopted the model results from a previous study (Wang et al., 2020), and incorporated them into this methodology. Please clarify if this is the case. As the authors aim to provide a framework for future assessments of water scarcity in other basins, a clear articulation of the methodology and data use is essential. Details such as databases used, temporal and spatial scales, could be presented in a table for greater clarity regarding the construction process and foundation of the method.

Our response: We would like to clarify that we indeed used output data of river exports of nitrogen from the MARINA-Nutrients model (version 2.0) published by Wang, et al. [1] for the Pearl River. The data for calculating river exports of nitrogen and its sources are all provided and summarized in the supplementary materials of Wang, et al. [1]. River export of nitrogen was provided at the sub-basin scale for total dissolved nitrogen (including inorganic and organic forms). We aggregated it to months and grids as explained in our supplementary Note 2. Following the reviewer's suggestion, we created a new Table (see /calculations and provides their sources and temporal and spatial scales Supplementary Table 4 in the revised supplementary materials) that lists the input data/parameters used in the different models.

Comment 3: *2. Uncertainty analysis or sensitivity analysis is crucial for the research of model simulation and future scenario forecasting. The manuscript or supplementary material should present some key calibration results from the MARINA model. I understand these results are adopted from a previous*

study, since the including of water quality based on MARINA model is the novelty of this study, it is important to discuss the uncertainty, not only the inherent uncertainty of the MARINA model. The authors should explore and discuss how these uncertainties might propagate into the final results of this method, and how they might impact predictions for the future.

Our response: We agree with the reviewer's comment. For this, we have added two new figures with key results from the MARINA-Nutrients model for the Pearl River (see new Supplementary Figures 2 and 3 in the revised supplementary information). In addition, we now elaborate more in the revised version of the manuscript on the uncertainties in the MARINA-Nutrients model results and how they could influence the main conclusions of our research related to future water scarcity assessments (see revisions in Lines 243-308 in the red color in the revised discussion section). We added the sensitivity analysis to better understand how uncertainties in the cost values and the different water scarcity thresholds could influence our main conclusions (See Lines 309-326).

We decided to do extra model runs to acknowledge uncertainties. We ran 12 times the model for testing the robustness of our main results to changes in the cost of water quality management options and to changes in water scarcity levels. We increased the costs of the water quality management options by +50% and +100% for the three scenarios (SSP2-RCP2.6, SSP2-RCP8.5 and SSP5-RCP8.5). We kept the costs of the water quantity management options as in the baseline run. We did this to better understand how uncertainties in the costs of the water quality management options could influence the main results of the paper that water quality management options are the most cost-effective to reduce future water scarcity. We also changed the water scarcity levels from medium (0.2) to high (0.4) and low (0.1). We did this to better understand how uncertainties in our water scarcity threshold could influence our main conclusions. The results indicate the robustness of our conclusions.

In the revised manuscript, we did:

- The sensitivity analysis setup is now available in the revised supporting information (see Supplementary Tables 5 and 6 in the revised supporting information);
- We added the description of the sensitivity set up in the revised methods (see Lines 514-524);
- We presented and discussed the results of the sensitivity analysis in the revised discussion section. Here we also elaborated on uncertainties and their influence on our main conclusions in the revised discussion section (see Lines 309-327).

Comment 4: *3. The manuscript refers to the use of the General Algebraic Modeling System (GAMS) for optimization. Can the optimization results be processed in conjunction with uncertainties from the MARINA model?*

Our response: The optimization model has been run in GAMS (a well-established and efficient software for mathematical programming) under three future climate and socio-economic scenarios to cover a wide range of future conditions. The model uses results from the MARINA-Nutrients model simulations. Thus, it is possible to process optimization results in conjunction with uncertainties from the MARINA-Nutrients model. The results of the optimization model are shown to be robust under the different scenarios, indicating that the potential uncertainties from the MARINA-Nutrients model would not substantially change the direction of the optimization results.

Comment 5: *4. The nitrogen legacy effect is crucial, which has been increasingly recognized and emphasized in global watershed studies. Can you discuss some potential implications of the legacy effect and its future impact on water quality scarcity.*

Our response: It is a good point. This comment is also related to Comment 3 above (see our response to that comment). We, of course, realize the importance of the legacy effects of nitrogen in river systems.

We agree with the reviewer that this aspect should be discussed in the manuscript. For this, we revised the discussion section by discussing the implication of the legacy effects of nitrogen on our results for future water scarcity (see revisions on Lines 243-256 in the red color in the revised discussion section).

Comment 6: *5. In Supplementary note 2, the MARINA-Nutrients model is described to calculate N concentrations annually, and the amount of dissolved nitrogen is assumed to be equal across all months (Line 57-60, Supplementary note). Such an assumption may introduce uncertainty when comparing and discussing monthly results (Fig. 2). The discussion on seasonal variations of water scarcity levels should consider the uncertainty introduced by this assumption.*

Our response: This comment is related to Comment 3 above. Please kindly see our response to Comment 3 above. We would like to highlight that we realize the uncertainties in our manuscript that are associated with the model scales and our aggregations. We did not properly discuss these aspects in our original manuscript. This was our omission. We do now address uncertainty in the revised manuscript (see revisions on Lines 243-327 in the red color in the revised discussion section). See our response to Comment 3 on the uncertainty and sensitivity analysis.

Reviewer #2

Comment 7: *Water scarcity is a global challenge, and has been increasingly concerned by both water managers and researchers during the past decade. Therefore, I believe the topic of the manuscript is interesting, and well in the scope of NC. However, I am not well convinced. Detailed comments were given as follows.*

Our response: We thank the reviewer for the evaluation of our manuscript and thoughtful comments provided. Water scarcity is a major global challenge and identifying cost-effective solution options to address it in highly water-stressed river basins under future climate and socio-economic scenarios should be relevant from both scientific and practical/policy perspectives. In our responses below, we explain how we addressed the comments of the reviewer and incorporated them into the revised manuscript.

Comment 8: *1) Title: The title is interesting. However, I am not well convinced. The study area of this study is the Pearl River Basin, a large watershed in south China. It is a regional study, rather than national or global study. Therefore, the conclusion (title) may not be true for other watersheds. In particularly, in north China, the water resources are not as rich as the study area (Pearl River Basin), water quality management options would be much more costly.*

Our response: The Pearl River Basin is expected to undergo several changes in the future, which are common for many river basins around the world such as urbanization, industrialization, and changing water availability. Therefore, the results from the application of our integrated modeling framework to the Pearl River basin could be useful for other river basins in the world undergoing similar conditions. Moreover, our results indicate that in river basins where water pollution is significant, it should be considered in the assessment of water scarcity as well as in the design of water management options for reducing water scarcity. Following the review's suggestion, we updated the title as follows: "Water quality management could cost-effectively halve future water scarcity in highly water stressed and polluted river basins". We hope that it is now more convincing.

Comment 9: *2) Abstract: authors claimed model coupling (water quality model with a cost-optimization procedure) as novel modeling approach. I am not fully agreed. Coupling a process-based model with a cost-optimization procedure has been published before, e.g., https://doi.org/10.1016/j.spc.2022.01.031.*

Our response: We thank the reviewer for the suggested paper that focuses largely on energy and the abatement of CO_2. Our focus is different. We focus on biogeochemical cycles of nitrogen (representing water quality), and couple that to other aspects of water scarcity. We see the point of the reviewer. However, we do believe that the way of how we coupled the water quality model (MARINA-Nutrients based on biogeochemical cycles, which was not in the suggested paper) with a cost-optimization procedure as well as the purpose of this coupling is very novel. To the best of our knowledge, there are no studies in the literature that investigated the cost-efficiency of water quantity and quality management options and identified cost-effective combinations of these options to reduce water scarcity under various future climatic and socio-economic scenarios using an integrated biophysical-economic modeling approach. Perhaps, we were not clear enough about this in our manuscript. We clarify the novel aspects in the revised manuscript (see revisions in the abstract on Lines 27-30 in the red color, Lines 67-79 in the red color in the revised introduction section and Lines 257-275 in the red color in the revised discussion section).

***Comment 10**: 3) Introduction: "To our knowledge, there is no study available using the cost-efficiency of management options to reduce water scarcity". I am not fully agreed with this statement. This issue has been investigated in previous publications, e.g., https://doi.org/10.3390/w14193056. Therefore, I suggest authors to better highlight the contribution of this study.*

Our response: Thank you for your thoughtful comment. This comment is related to Comment 9 above (please kindly see our response to that comment). The suggested paper by the reviewer is interesting but largely focuses on water as a quantitative input. We add water quality in our water scarcity assessment and consider the complex biogeochemistry (for nitrogen through the MARINA-Nutrients model) that are influenced by human activities on the land (through the MARINA-Nutrients model) under the scenarios with low and high global warming. We couple those aspects in the water scarcity indicator that considers quality (nitrogen in our case) and quantity (water availability) and with cost-optimization, allowing us to identify the combination of cost-effective solutions for both quality (reducing nitrogen pollution from agricultural fields and better wastewater treatment) and quantity (saving, transfer, and storage technologies). This is very different from the suggested paper. We clarified the knowledge gap in the revised introduction (see the changes in red on 64-93).

***Comment 11**: 4) Line 261: This paragraph discussed the model uncertainties from model structure. However, the data from climate and socioeconomic scenarios, and the costs of water quantity and quality management options (Supplementary Table 3) may be another uncertainty sources.*

Our response: This comment is about uncertainties and related to Comment 3 above (please see our response to that comment). We agree with the reviewer that the data also have uncertainties. In our revised discussion, we discussed uncertainties in data for climate and socioeconomic scenarios and the costs of the management options (see revisions in red on Lines 276-326).

***Comment 12**: 5) Line 275: The model included only N as a water quality parameter. Why not add P as another water quality parameter. Compared to N concentration, P concentration is more highly concerned by water managers due to its highly related to lake eutrophication and harmful algal blooms.*

Our response: The reviewer has a good point. P is important when considering lake pollution control against harmful algal blooms. In fact, N and P are interconnected and should be considered together when managing water quality. We would like to clarify that our manuscript does not focus on lakes. We focus on rivers in the entire Pearl Basin. We selected N because of N impacts on several sectors including agriculture, domestic, and nature (eutrophication processes). Including P is a major step and would result in a separate paper. In the revised manuscript, we clarified the choice of N (in dissolved forms) in our water scarcity analysis (see revisions in red on Lines 328-330), discussed the implication of

having N without P on our main results (see revisions in red on Lines 332-334) and added P as a recommendation to include in future studies (see revisions in red on Lines 330-334).

***Comment 13**: 6) Cost calculation: I guess the investigated results are very sensitive to the cost calculation parameters (i.e., Supplementary Table 3). I fully understand it is extremely challenging to determine these parameter values. However, I believe considerable efforts are needed to convince the use of these values. For example, the cost of water transport is 0.02-0.38. However, I am not sure if it is able to carry our water transfer projects in these areas. I do not have too much experiences at the study area (Pearl River Basin), and was not able to evaluate the reasonability of these parameters. Moreover, references for these values would be helpful.*

Our response: We agree with the reviewer that the results of cost-optimization models might be sensitive to the cost parameters. However, in our study, we believe that the relative differences among the costs of the different water management options are reasonable and therefore we do not expect significant changes in the direction of our results. Determining the costs of the different water management options in the Pearl River is challenging as also indicated by the reviewer. Therefore, we have used data from other river basins or regions with similar climate and economic conditions based on an extensive literature review (see revisions in red in our revised discussion section).

For the water transport projects, Rogers et al.[2] indicated that water transport is a common water reallocation practice in China, assessing the implementation of China's South-North water transfer project. The cost of water transfer depends on the total distance the water is transferred. The distances are calculated as the accumulated straight-line distance between the centers of the consecutive cities from Denghzou to Beijiang. The cost of water transfer varies between 0.02 and 0.38 €/m^3 depending on the distance between cities and is given by Pohlner [3]. The estimation of the distance between cities and the costs of water transport are explained in Supplementary Note 6 and Supplementary Table 1.

About the sensitivity of the costs and data: see our response to Comment 3 above. We would like to clarify that we did the sensitivity analysis to better understand how uncertainties in costs and water scarcity levels would influence our main conclusions. The results of the sensitivity analysis confirmed the robustness of our conclusions (see Lines 309-327 in the revised manuscript).

***Comment 14:** 7) Model validation: The authors stated that "The model has been validated and evaluated by Wang, et al. 40". i) I think some brief description on the validation results (validated data, model fit, etc.) are needed. ii) I have a quick scan on the reference. To be honest, I am not very satisfied on the validated results of this study. In particularly, the reference compared the estimated nutrient load (annual) with measurement. This would achieve a high model fit compared to the comparison based on nutrient concentration.*

Our response: We agree with the reviewer that model validation is important. We also feel that we did not spend enough words on this. In our manuscript, we used the MARINA-Nutrients model. The modeling approach has been evaluated using a "building trust" approach[4]. This approach includes model validation, sensitivity analyses, and comparisons with other studies. This means that our model evaluation is based not only on validation but also on other aspects. In addition, the model was evaluated across Chinese rivers[1,5-7] including seasons[8] and lakes[9-11]. Furthermore, the model has been applied to other regions in the world and was also evaluated[12-14]. We clarified this in the revised methods (see revisions in red in the Methods section). In addition, we performed a new sensitivity analysis (See Lines 309-327 in the revised manuscript).

Reviewer #3

Comment 15: *This paper integrates established approaches to measuring water quantity and water quality to develop a single, integrated quantity/quality characterization of water scarcity in China's Pearl River Basin. The authors project both water quality (using nitrogen concentration as the sole parameter of interest) and water quantity (river discharge) at the 0.5-km2 grid cell level to 2050 under three different future climate/socioeconomic conditions. They then estimate the costs and benefits (in terms of reduced water scarcity) of various mitigation approaches, including agricultural water quality and irrigation efficiency measures, urban residential water efficiency investments, and water storage and transport infrastructure investments. They conclude that measures to reduce N inputs from the agricultural sector (the "water quality" options) are more cost-effective approaches to reducing the future occurrence of water scarcity conditions in the Pearl River basin.*

Our response: We thank the reviewer for the positive evaluation of our manuscript and the thoughtful comments provided. In our responses below, we explain how we addressed the comments of the reviewer and incorporated them into the revised manuscript.

Comment 16: *This is interesting work, and it represents a contribution relative to the prior literature. I have some more specific comments for the authors, which I hope will be useful as they continue to revise their work.*

Our response: We thank the reviewer for recognizing the contribution of our work to current literature.

Comment 17*: 1. The paper does not provide enough description of the water quality criterion used to determine whether each part of the Basin has water of sufficient ambient quality for its designated use. Lines 122-124 suggest that areas with an average concentration above 1.0mg/l of N "do not meet the N quality standard...for all sectors." In the United States, the maximum contaminant level (MCL) for nitrite in finished drinking water is 1mg/l. The MCL for nitrate in finished drinking water is 10mg/l. The WHO standard for nitrate in finished drinking water is even higher (50mg/l as NO3, or 11.3mg/l as NO3-N). The paper does not provide enough background on water quality in the Basin to help the reader understand whether nitrate or nitrite is the greater concern, and how they chose the 1mg/l standard for ambient concentrations in the river. This seems very stringent - can the authors explain this better, and if their threshold is too low, how do the results change if it is raised?*

Our response: The reviewer is asking for more clarification on how we derived the water quality standard of 1.0 mg/L for nitrogen. In fact, we did not clarify this enough. We thank the reviewer for giving us this opportunity to add the clarification. We derived the water quality standard from the Chinese water quality classes because our study area was the Pearl River located in China. In China water quality standards are described in the national environmental quality standards for surface water [15]. Water quality standards for nitrogen concentrations are respectively 1.0, 1.5, and 2.0 mg per litter (mg/l) for the domestic, industrial, and agricultural sectors[15]. In this study, the water quality standard of nitrogen for the domestic sector of 1.0 mg/l is used to calculate the water that is needed for diluting water to sufficient water quality (see Supplementary Box 1 for the equations). This choice was made because the domestic sector has the most stringent standard for N compared to the other two sectors. This implies that if the standard for the domestic sector is met, then water quality is also sufficient for the industrial and agricultural sectors. Selecting the strictest standards has also benefits for the other sectors. Nitrogen is considered as total dissolved nitrogen that consists of dissolved inorganic and organic forms. We clarified those aspects in the revised method section (see revisions in red in the Methods section). In addition, we did a new sensitivity analysis in which we changed the water scarcity levels. Our main conclusions did not change. This means that when we assumed a low threshold for

water scarcity (0.2), the main messages on the cost-effective management options remained valid. The same results were obtained for the high level of water scarcity (0.4). This indicates the robustness of the main conclusions of our paper (see lines 309-327 in the revised manuscript).

Comment 18: *2. I realize that the water scarcity measure, itself, is the subject of a separate, published paper, and it is partially explained in the supplemental information. However, I found it jarring to read the results section, which simply applies the water scarcity measure without explaining to the reader what it is - how it is calculated, that it is unitless, and how we should interpret it. Relatedly, please define the terms in the denominator of the water scarcity measure equation (1) in the supplemental information.*

Our response: We agree that we should have described the water scarcity indicator better. We now do this on Lines 362-397 in the revised manuscript. We checked the definitions of the numerators in all equations and corrected where it was needed (see revisions in red in revised Supporting Box 1).

Comment 19*: 3. Figure 2 is important to the paper, but I found it very difficult to read. Here are some suggestions:*
(a) Either drop 2(a), or explain how to interpret this sub-figure in both the figure notes and in the text of the paper. I found that portion of Fig. 2 totally un-interpretable.
(b) I found it confusing that N is measured in red and water quantity in blue in 2(b), but the opposite color scheme is applied in 2(c).
(c) In 2(b) and (c), it seems clear that there is no meaningful change in river discharge, at least at the basin scale, under the three different RCP/SSP scenarios. This bears mentioning in the paper. Given this fact, it is not terribly surprising that addressing the water quality problems (which do change substantially under the three scenarios) are really the only cost-effective path to mitigating scarcity.

Our response:

(a) The reviewer has a good point. In the revised manuscript we now explain how to interpret the sub-figure 2(a) in the figure note (see revisions in red in the caption of Figure 2).
(b) The color of each parameter used in Figure 2 is explained in the figure note in the revised manuscript. In 2(a) and 2(c) the black color represents water scarcity including only water quantity and the grey color shows water scarcity including both water quantity and quantity. In 2(b) and 2(c), the blue schemes represent river discharge whereas the red represents the water quality parameter N.
(c) We now mention that there is no meaningful change in river discharge and the water scarcity issue is strongly associated with water quality rather than quantity on lines 128-131 in red.
The cost-efficiency of water quantity and quality options depends on the cost and the abatement potential of each option. For example, water storage (water quantity option) could increase river discharge by 212 billion m^3 under the RCP2.6-SSP2 scenario, reducing water scarcity by 34%. This option is the most efficient alternative in reducing water scarcity, but it has high investment and operation costs (of about 153 $ billion) which lead to a cost-efficiency of about 4.5 $ billion/% of water scarcity abatement. However, treating human waste (water quality option) could reduce N pollution by 128 Kton under the RCP2.6-SSP2 scenario, reducing water scarcity by 25% at a cost of about 0.00003 $ billion. This option has a cost-efficiency of about 0,000001 $ billion/% of water scarcity abatement. As a result, treating human waste is more cost-effective than water storage.

Comment 20*: 4. Figure 3 is also important to the paper. But given the description in Supplementary Note 5 of how the MACC were calculated (dividing TC by the % of water scarcity abatement achieved with each option), these are not marginal cost curves. They are average cost curves. Marginal cost curves would not include fixed costs.*

Our response:

Yes, you are right. The marginal cost curve represents the additional cost incurred by producing one more unit of a good or service. It is a fundamental concept in microeconomics and is used by economic agents to make production and pricing decisions. In economic terms, it is the cost of producing an additional unit at any given production level. The marginal cost is equal to the derivative of the total cost (zero for fixed costs as these are independent of production levels).

The marginal abatement cost curve is used in environmental economics and policy, particularly in the context of pollution control and greenhouse gas emissions reduction. It represents the cost of reducing one additional unit of pollution or emissions. The marginal abatement cost is calculated as the change in total cost divided by the change in the level of pollution reduction.

In our study we estimate the marginal abatement cost (i.e., the cost-efficiency of the measure and the abatement potential) not the marginal cost.

We would like to clarify that the marginal abatement cost curve (MACC) was first used by McCarl and Schneider (2000) [16] to analyze mitigation policies in US agriculture. Subsequently, it has been used in different studies at global, regional, and national levels [16-19]. The MACC is a tool for policy analysis that brings a wide range of information about mitigation options and shows the potential abatement and the associated costs for different alternatives. This information reveals which are the most cost-effective policy interventions to facilitate the exchange between scientific studies and policy decision-making.

The marginal cost of abatement can be calculated in various ways. Vermont and De Cara (2010)[20] divides MACCs into three main types based on the methodology used to derive the curves: (i) bottom-up cost-engineering; (ii) micro-economic modeling, with exogenous prices; (iii) regional/sectoral supply-side equilibrium models. Macleod et al.[21] indicated that the MACC derived from modeling are often smooth curves, while those based on bottom-up cost engineering approaches are more often represented as a series of discrete bars, each of which represents a mitigation measure. The width of each bar represents the pollution reduction, while the height of the bar shows the cost-efficiency of the measure. The area under each bar is equal to **the total cost of the measure** (in other words the cost-efficiency = the total costs of option/abatement).

In our study, the MACC was developed following a bottom-up engineering cost approach. In this approach, information on the one-off and recurring costs of a range of water management options and their effect on water saving and N pollution is collected. These data are used to calculate (a) the annual cost of each option, (b) the amount of water scarcity reduced each year, and (c) the cost-efficiency, i.e., the cost per unit of water scarcity abatement. The results for each option are then plotted, usually in order of increasing cost-efficiency. So, the MACC shows the cost of reducing water scarcity by one unit (expressed in % of water scarcity abatement).

We include more information about the MACC analysis in the revised manuscript on lines 466-473 and in supplementary note 5.

Comment 21: *5. The discussion section is too long and repetitive of text that appears earlier in the paper. It also introduces some new results (in lines 223-232, for example, describing specific agricultural water quality management options). I suggest moving that discussion to the results section, and otherwise substantially shortening the discussion.*

Our response: Thank you for your suggestion. We have moved this discussion part to the results section and improved the discussion based on the reviewers' comments. We also improved the discussion

section by elaborating more on the influence of uncertainties on the main conclusions of this paper (See Lines 309-327 in the revised discussion section).

References used in this response letter

1 Wang, M., Kroeze, C., Strokal, M., van Vliet, M. T. & Ma, L. Global change can make coastal eutrophication control in China more difficult. *Earth's Future* **8**, 1-19 (2020).

2 Rogers, S. *et al.* An integrated assessment of China's South-North Water Transfer Project. *Geogr. Res.* **58**, 15, doi:10.1111/1745-5871.12361 (2020).

3 Pohlner, H. Institutional change and the political economy of water megaprojects: China's south-north water transfer. *Glob. Environ. Change-Human Policy Dimens.* **38**, 205-216, doi:10.1016/j.gloenvcha.2016.03.015 (2016).

4 Strokal, M. River export of nutrients to the coastal waters of China: the MARINA model to assess sources, effects and solutions (PhD thesis). *Wageningen University, Wageningen, The Netherlands* 235pp (2016).

5 Strokal, M., Kroeze, C., Wang, M., Bai, Z. & Ma, L. The MARINA model (Model to Assess River Inputs of Nutrients to seAs): model description and results for China. *Science of the Total Environment* **562**, 869-888 (2016).

6 Wang, M. *et al.* Accounting for interactions between Sustainable Development Goals is essential for water pollution control in China. *Nature communications* **13**, 1-13 (2022).

7 Chen, X. *et al.* In-stream surface water quality in China: A spatially-explicit modelling approach for nutrients. *Journal of Cleaner Production*, 130208 (2021).

8 Chen, X. *et al.* Seasonality in river export of nitrogen: A modelling approach for the Yangtze River. *Science of The Total Environment* **671**, 1282-1292, doi:https://doi.org/10.1016/j.scitotenv.2019.03.323 (2019).

9 Yang, J. *et al.* Nutrient losses to surface waters in Hai He basin: A case study of Guanting reservoir and Baiyangdian lake. *Agricultural Water Management* **213**, 62-75 (2019).

10 Yang, J. *et al.* What is the pollution limit? Comparing nutrient loads with thresholds to improve water quality in Lake Baiyangdian. *Science of the Total Environment* **807**, 150710 (2022).

11 Yang, J. *et al.* Sources of nitrogen in reservoirs of the Haihe basin (China) 2012–2017. *Journal of Environmental Management* **345**, 118667 (2023).

12 Strokal, M. *et al.* Urbanization: an increasing source of multiple pollutants to rivers in the 21st century. *Urban Sustainability* **1**, 24 (2021).

13 Li, Y. *et al.* Multi-pollutant assessment of river pollution from livestock production worldwide. *Water research* **209**, 117906 (2022).

14 Goshu, G., Strokal, M., Kroeze, C., Koelmans, A. A. & de Klein, J. J. M. Assessing seasonal nitrogen export to large tropical lakes. *Science of The Total Environment* **731**, 139199, doi:https://doi.org/10.1016/j.scitotenv.2020.139199 (2020).

15 MEE. Environmental Quality Standards for Surface Water (GB3838-2002). (2002).

16 McCarl B.A. & U.A., S. U.S. Agriculture's Role in a Greenhouse Gas Emission Mitigation World: An Economic Perspective. . *Appl. Econ. Perspect Policy. 2000;22:134–159. doi: 10.1111/1058-7195.t01-1-00011.* (2000).

17 Albiac, J., Kahil, T., Notivol, E. & Calvo, E. Agriculture and climate change: Potential for mitigation in Spain. *Sci. Total Environ.* **592**, 495-502, doi:10.1016/j.scitotenv.2017.03.110 (2017).

18 MacLeod, M. *et al.* Developing greenhouse gas marginal abatement cost curves for agricultural emissions from crops and soils in the UK. *Agric. Syst.* **103**, 198-209, doi:10.1016/j.agsy.2010.01.002 (2010).
19 Pellerin, S. *et al.* Identifying cost-competitive greenhouse gas mitigation potential of French agriculture. *Environ. Sci. Policy* **77**, 130-139, doi:10.1016/j.envsci.2017.08.003 (2017).
20 Vermont, B. & De Cara, S. How costly is mitigation of non-CO₂ greenhouse gas emissions from agriculture? A meta-analysis. *Ecol. Econ.* **69**, 1373-1386, doi:10.1016/j.ecolecon.2010.02.020 (2010).
21 MacLeod, M., Eory, V., Gruere, G. & Lankoski, J. Cost-effectiveness of Greenhouse Gas Mitigation Measures for Agriculture: a Literature Review. Report No OECD Food, Agriculture and Fisheries Papers, No. 89. OECD Publishing, Paris. (2015).

REVIEWER COMMENTS

Reviewer #1 (Remarks to the Author):

Thank you for the response from the authors. The addition of sensitivity analysis and uncertainty analysis, along with the discussion on legacy effects, addresses some of the concerns I raised. However, I believe further improvements are needed in the discussion regarding legacy effects. Firstly, the impacts of legacy effects often observed in inter-annual variations, with an increasing body of research suggesting that legacy nitrogen can affect the environment for years to decades. For example, the ELEMENT model simulate annual riverine nitrogen exports and their potential legacy effects, as opposed to modeling intra-annual variability (Chen et al., 2018; Van Meter et al., 2018; Basu et al., 2022). Secondly, it was mentioned that changes in water bodies due to past land use can intensify the impacts of climate-induced droughts or floods—resulting in increased water scarcity in some regions and excess water in others, thus impacting both water quality and availability. However, this discussion predominantly focuses on water quantity. However, the nutrient legacy effect will directly affect water quality significantly, where nutrient storages in soil and groundwater act as sources and are gradually released into surface water bodies through hydrological processes.

References
Chen, D., Shen, H., Hu, M., Wang, J., Zhang, Y. and Dahlgren, R.A., 2018. Legacy nutrient dynamics at the watershed scale: principles, modeling, and implications. Advances in agronomy, 149, pp.237-313.
Van Meter, K.J., Van Cappellen, P. and Basu, N.B., 2018. Legacy nitrogen may prevent achievement of water quality goals in the Gulf of Mexico. Science, 360(6387), pp.427-430.
Basu, N.B., Van Meter, K.J., Byrnes, D.K., Van Cappellen, P., Brouwer, R., Jacobsen, B.H., Jarsjö, J., Rudolph, D.L., Cunha, M.C., Nelson, N. and Bhattacharya, R., 2022. Managing nitrogen legacies to accelerate water quality improvement. Nature Geoscience, 15(2), pp.97-105.

Reviewer #2 (Remarks to the Author):

I have some comments on the authors' response.
Title: My previous comment was "It is a regional study, rather than national or global study.". Therefore, from my perspective, the revised version is not satisfied. I suggested adding "Pearl River Basin" into the title to specific the location of the case study.
Comment 12: Authors stated that "Including P is a major step and would result in a separate paper.". Actually, I do not agree this activity. From my perspective, the including of P would probably achieve different conclusions. Therefore, I suggested adding P into this study.

Reviewer #3 (Remarks to the Author):

The authors have addressed my comments from the previous round of review.

Response to comments on the paper "Water quality management could cost-effectively halve future water scarcity in highly water stressed and polluted river basins".

Foremost, we would like to thank the three reviewers for the valuable comments that have helped us to improve substantially the quality of the paper. We also thank the editor for the opportunity to revise and resubmit our manuscript. Two important points were raised by the reviewers: (1) the legacy effects, and (2) adding phosphorus to the model calculations. We believe that the second point is a major effort and could easily result in a separate new paper. We followed the suggestion of the editor to include a discussion on phosphorus and highlight that this work focuses on nitrogen solely. We have responded to all comments as below. The revisions we have made are highlighted in the revised manuscript in red.

Reviewer #1 (Remarks to the Author):

Comment 1: *Thank you for the response from the authors. The addition of sensitivity analysis and uncertainty analysis, along with the discussion on legacy effects, addresses some of the concerns I raised.*

Our response: Thank you for your feedback. We are pleased to hear that the addition of sensitivity and uncertainty analysis, as well as the discussion on legacy effects, have helped to address some of the reviewer's concerns. We appreciate the reviewer's valuable input, and we address the additional comment below.

Comment 2: *However, I believe further improvements are needed in the discussion regarding legacy effects. Firstly, the impacts of legacy effects often observed in inter-annual variations, with an increasing body of research suggesting that* **legacy nitrogen can affect the environment for years to decade**s. For example, the ELEMENT model simulates annual *riverine nitrogen exports and their potential legacy effects, as opposed to modeling intra-annual variability (Chen et al., 2018; Van Meter et al., 2018; Basu et al., 2022). Secondly, it was mentioned that changes in water bodies due to past land use can intensify the impacts of climate-induced droughts or floods—resulting in increased water scarcity in some regions and excess water in others, thus impacting both water quality and availability. However, this discussion predominantly focuses on water quantity. However, the nutrient legacy effect will directly affect water quality significantly, where nutrient storages in soil and groundwater act as sources and are gradually released into surface water bodies through hydrological processes.*

References

Chen, D., Shen, H., Hu, M., Wang, J., Zhang, Y. and Dahlgren, R.A., 2018. Legacy nutrient dynamics at the watershed scale: principles, modeling, and implications. Advances in agronomy, 149, pp.237-313.
Van Meter, K.J., Van Cappellen, P. and Basu, N.B., 2018. Legacy nitrogen may prevent achievement of water quality goals in the Gulf of Mexico. Science, 360(6387), pp.427-430.
Basu, N.B., Van Meter, K.J., Byrnes, D.K., Van Cappellen, P., Brouwer, R., Jacobsen, B.H., Jarsjö, J., Rudolph, D.L., Cunha, M.C., Nelson, N. and Bhattacharya, R., 2022. Managing nitrogen legacies to accelerate water quality improvement. Nature Geoscience, 15(2), pp.97-105.

Our response: Thank you for your insightful comment on the discussion regarding legacy effects and land use change. We appreciate your suggestions for further improvements of this aspect.

Regarding the legacy effects, we fully agree with reviewer's comment. As you rightly indicated, legacy effects may influence nutrient cycling, and thus water quality. For example, nutrients can accumulate in soils and groundwater and then continue to be released to surface water systems over time. As a result, more nutrients could potentially enter the surface water systems (which is not explicitly accounted for in our modeling approach in this paper). However, the speed of releasing nutrients from the soil and groundwater systems also depends on soil characteristics (e.g., clay can accumulate more than sand), hydrology (e.g., higher release of nutrients to the water system in basins with high precipitation surplus) and chemical characteristics (e.g., phosphorus has much more binding abilities to soil particles than nitrogen). Therefore, the legacy effects across inter-annual variations entail that past actions or events, such as historical land use practices or pollution inputs have long-term consequences on water quality.

The legacy effects are often observed as consistent patterns or trends that persist from one year to the next, despite changes in environmental conditions or management practices. For example, nutrient legacies from previous fertilizer applications or land management practices can persist in the soil and affect nutrient availability to plants over multiple growing seasons. Additionally, legacy effects have substantial implications for water quality, as nutrients stored in soil and groundwater can serve as sources of pollution, impacting surface water bodies through hydrological processes and posing risks to human health.

Phosphorus has generally higher binding abilities than nitrogen. This implies that phosphorus can stay even longer in the system and can be released into water over time for decades. In one of our earlier studies[1], we assessed the legacy effect of phosphorus in the soil solution and soil solid phases on the river export of phosphorus for river basins globally. One of our findings was that phosphorus was released from the soil continuously into rivers even after stopping the use of fertilizers on land. However, river export of phosphorus was somewhat affected because the dominant source of phosphorus in many rivers was the sewage system. For river exports of nitrogen, this may be different and likely also depends on the dominant sources. In our study area, the dominant sources differ among the sub-basins[2,3]. In the downstream sub-basins, the sewage systems are important because of the urbanization trends. In those sub-basins, the biases in our water quality simulation associated with legacy effects is somewhat lower. In the up-middle stream sub-basins, agriculture plays an important role. Here we may under- or over-estimate pollution levels depending on soil characteristics and hydrology. Furthermore, we would like to also highlight that some nitrogen can easily leave the system via denitrification processes and thus it is perceived as more mobile (less accumulation) than phosphorus. As a result, nitrogen to phosphorus ratios may also change affecting water quality[4].

In our revised manuscript, we further extended the discussion on legacy effects, acknowledged that we do not account for this in our modelling simulations, and added more information and references as suggested by the reviewer to provide a holistic understanding of the legacy effects and the interactions between land use, hydrology, and water quality (please see revisions in the discussion section on Lines 284-320).

Reviewer #2 (Remarks to the Author):

Comment 3: *I have some comments on the authors' response.*

Our response: We thank the reviewer for the evaluation of our manuscript and thoughtful comments provided. In our responses below, we explain how we addressed the comments of the reviewer and incorporated them into the revised manuscript.

Comment 4: *Title: My previous comment was "It is a regional study, rather than national or global study.". Therefore, from my perspective, the revised version is not satisfied. I suggested adding "Pearl River Basin" into the title to specific the location of the case study.*

Our response: Following the reviewer's suggestion, we updated the title as follows: "Water quality management could cost-effectively halve future water scarcity: a case study in the Pearl River Basin".

Comment 5: *Authors stated that "Including P is a major step and would result in a separate paper." Actually, I do not agree this activity. From my perspective, the including of P would probably achieve different conclusions. Therefore, I suggested adding P into this study.*

Our response: Thank you for your feedback. We understand your perspective regarding the inclusion of phosphorus (P) in this study and the potential impact it could have on the conclusions. However, adding P is a major effort and we still believe this could result in a separate new paper. It will require re-structuring of the modeling approach and will likely result in a new modeling system. Considering the editor's suggestion, we decided to discuss the implications of excluding P in our study and propose this as the next step (please see revisions in the discussion on Lines 296-320).

Reviewer #3 (Remarks to the Author):

Comment 6: *The authors have addressed my comments from the previous round of review.*

Our response: We thank the reviewer for the positive evaluation of our manuscript. We are pleased to hear that the comments from the previous round of review have been adequately addressed.

References:

1 Strokal, M. & de Vries, W. Dynamic modelling of phosphorus export at river basin scale based on Global NEWS. *Report 2321, Alterra Wageningen UR, Wageningen, the Netherlands*, 100 pp. (2012).

2 Wang, M. *et al.* Accounting for interactions between Sustainable Development Goals is essential for water pollution control in China. *Nature communications* **13**, 1-13 (2022).

3 Strokal, M. *et al.* Increasing dissolved nitrogen and phosphorus export by the Pearl River (Zhujiang): a modeling approach at the sub-basin scale to assess effective nutrient management. *Biogeochemistry* **125**, 221-242 (2015). https://doi.org:10.1007/s10533-015-0124-1

4 Yin, K., Harrison, P. J., Broom, M. & Chung, C. Ratio of nitrogen to phosphorus in the Pearl River and effects on the estuarine coastal waters: nutrient management strategy in Hong Kong. *Physics and Chemistry of the Earth, Parts A/B/C* **36**, 411-419 (2011).
